# A national model for estimating United States public land visitation

**DOI:** 10.1038/s41598-025-26926-w

**Published:** 2025-11-28

**Authors:** Nathaniel H. Merrill, Samantha G. Winder, Dieta R. Hanson, Spencer A. Wood, Eric M. White

**Affiliations:** 1 Matunuck Research Group, Matunuck, RI USA; 2 Outdoor Recreation & Data Lab, University of Washington, Seattle, WA USA; 3 U.S. Department of Agriculture Forest Service, Pacific Northwest Research Station, Portland, OR USA

**Keywords:** Park visitation, Digital mobility data, Outdoor recreation, Federally managed lands, Environmental social sciences, Environmental economics

## Abstract

Public land management relies on accurate visitor counts in order to understand and mitigate environmental impacts and to quantify the value of ecosystem services provided by natural areas. We build and test predictive visitation models suitable for publicly-managed parks, open space, and other protected lands based on multiple sources of digital mobility data including geotagged posts to social media platforms, community science observations, and a mobile device location dataset from a commercial vendor. Using observational visitation data series from the United States’ National Park Service, Forest Service, and Fish and Wildlife Service, we quantify the accuracy of statistical models to predict on-the-ground visitation using individual and combined sources of mobility data alongside other covariates. We find the predictive models performed best in settings where some on-site visitation data can be integrated into the models. On-site visitation data helps to account for meaningful differences in modeled relationships both within and across the three agencies considered. We find variation in the usefulness of the digital mobility data sources, with models combining multiple data sources outperforming those using a single source, including those based solely on mobile device locations. We discuss the practical implications of these findings as well as paths forward to improve visitation estimation on public lands.

**Supplementary Information:**

The online version contains supplementary material available at 10.1038/s41598-025-26926-w.

## Introduction

Visitation estimates are critical for informing a wide range of questions about how to effectively manage public lands – from quantifying the economic contributions of outdoor recreation^1,2^, to the impacts of diminished air quality^3^, and events like oil spills and wildfires on park and beach usage^4–6^. As a result, most developed countries spend considerable resources developing sampling designs and counting visitors on public lands^2,7–9^. While these programs produce incredibly valuable data, the logistical challenges and expense of counting visitors using on-the-ground methods means monitoring programs rarely produce comprehensive estimates^10^, leading to pleas for more and better data coming from management agency staff^11^, academics^12,13^, and politicians^14^.

One approach to overcoming data limitations is to build predictive models for estimating visitation at many locations, including sites lacking any on-the-ground visitation counts. Models typically rely on the functional relationship between empirical visitation estimates and factors such as site characteristics, weather conditions, and nearby populations^15,16^. Recent research has begun evaluating whether predictive visitation models benefit from the addition of predictors derived from widely available digital mobility datasets^17–19^. Digital mobility data are locations of visitors derived from geolocated content shared on social media platforms and mobile device locations, among other sources^20^. To date, the limited evidence suggests that digital mobility data are informative predictors of visitation based on correlations^21–26^. Models incorporating a mix of functional variables and digital mobility data sources provide one potentially effective approach for estimation at individual sites and across land management units, according to comparison against on-the-ground visitation data^20,26^. Furthermore, the best-performing visitation models to date are ones that incorporate predictors from multiple mobility data sources^10,20,21,27^.

Studies have suggested that it may be feasible to construct generalizable visitation models that can be applied in estimation over large spatial and temporal scales, but this has not been tested. No study to date has evaluated alternative approaches for estimating recreation use at a national scale, across units managed by different entities. Consequently, we do not know which digital mobility data sources, or what combination of sources, are most useful for developing more general predictive visitation models. Furthermore, it is unclear how visitation models should be parameterized and applied in practice and the accuracy of the visitation estimates derived from models is not fully known.

In this study, we construct national-scale models of monthly visitation to individual recreation units managed by three United States federal agencies: the National Park Service (NPS), the Forest Service (USFS), and Fish and Wildlife Service (USFWS). We use on-the-ground visitation data and multiple predictors including five types of digital mobility data. We address three applied research questions (RQ): (1) what is the relative contribution of different types of digital mobility data to the performance of models that estimate visitation, (2) how transferable are the models among the three federal agencies, and (3) can the models be used to estimate visitation for new locations or unstudied time periods? This research is motivated by the practical application of using models built with digital mobility data for monitoring visitation to public lands and waters. We evaluate predictive models using cross-validation routines that correspond to potential applied uses of these models.

## Results

### Scatter plots and correlations

**Fig. 1:**
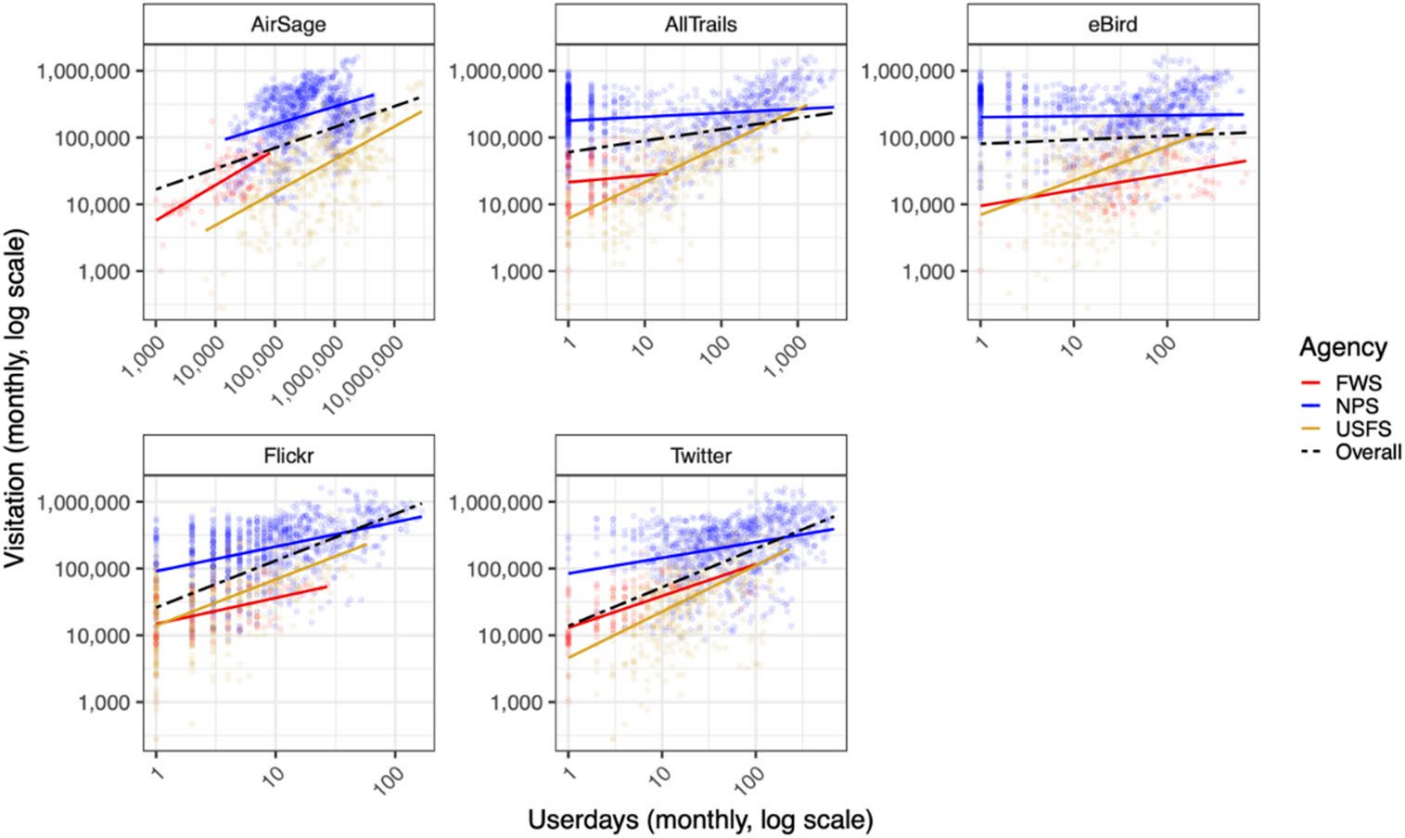
Scatter plots of each mobility data source against each agency’s observed visitation series with a linear regression line. Correlation values for each mobility data source are shown in Fig. 2 and listed in the Supplementary Materials (Table A6).

The overall correlation (all agencies combined) between each mobility data source and observed visitation ranged from a low of 0.07 for eBird to a high of 0.59 for Flickr (Fig. 1). Looking at each agency individually, the USFS generally had the highest overall correlations (ranging from 0.44 for eBird to 0.73 for AllTrails) and the NPS had the lowest correlations (from 0.03 for eBird to 0.43 for Flickr).

**Fig. 2:**
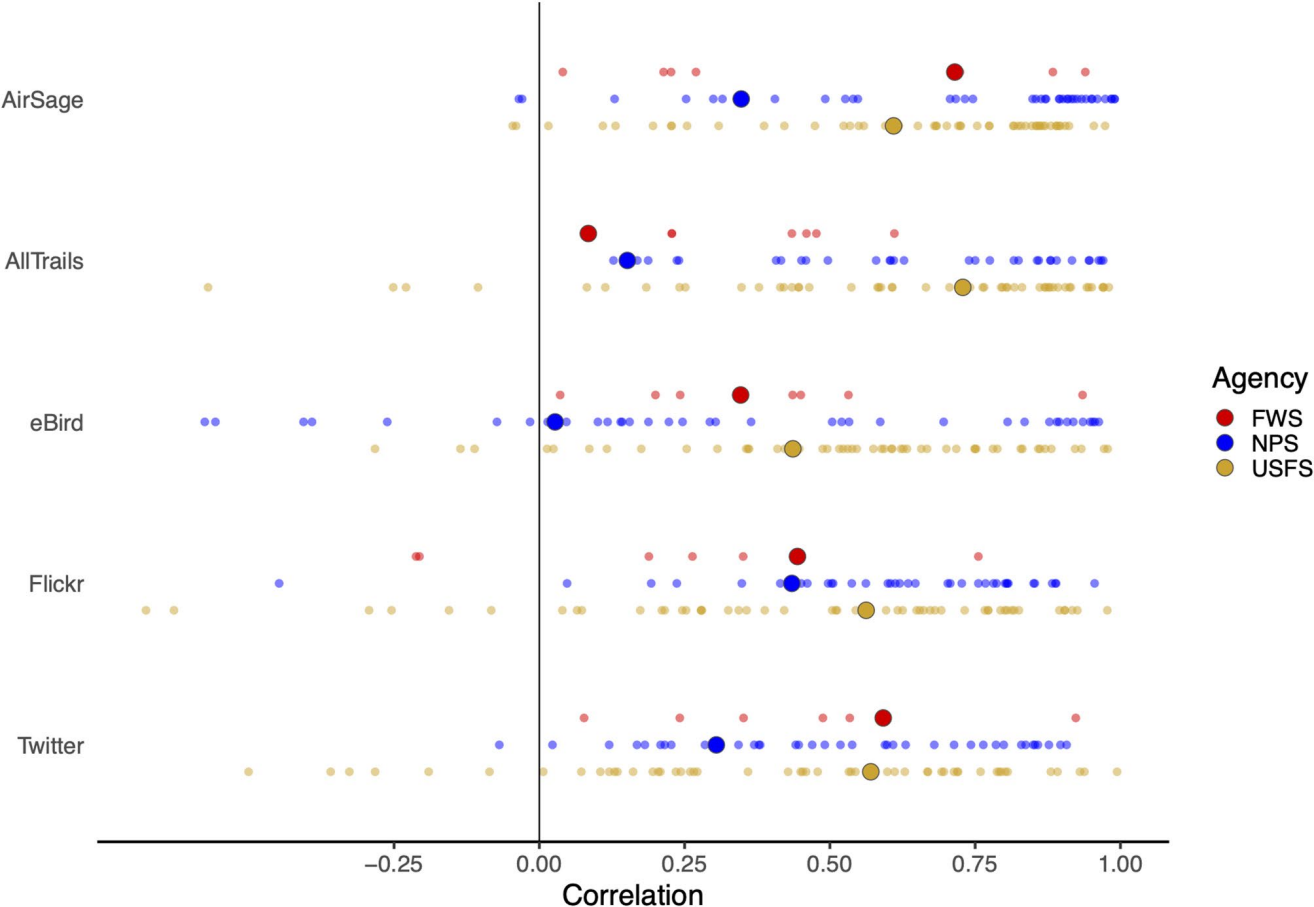
Pearson’s correlation between each mobility data source and observed monthly visitation at each unit (small dots) and overall for each agency (large dots). The overall correlation values correspond to the fit of the lines in Fig. 1.

When looking at correlations for units within each agency, there was relatively high variability (Fig. 2). For example, within the NPS, correlations between the AirSage data and observed visitation was less than zero at some units (−0.03 and −0.04 for Valley Forge National Historical Park and Rock Creek Park, respectively) but as high as 0.99 for others (Glacier National Park and Mount Rushmore National Memorial). Figures showing variation by year and agency are in the Supplementary Materials.

### Regression

**Table 1:** Base model regression results, from fitting the entire dataset. For the factor variables, the references are: month [1], region [Great Plains], year [2018], and agency [NPS].

Predictors	Estimates	CI	*p*
(Intercept)	2.98	1.41–4.54	< 0.001
AirSage	0.54	0.49–0.60	< 0.001
Flickr	0.15	0.11–0.18	< 0.001
Twitter	0.16	0.12–0.20	< 0.001
AllTrails	0.17	0.14–0.21	< 0.001
eBird	0.04	0.01–0.08	0.012
temperature °C	0.01	0.01–0.02	0.001
population within 30mi	0.10	−0.01–0.21	0.089
population in unit	−0.27	−0.34 – −0.20	< 0.001
region [Intermountain]	0.51	−0.24–1.26	0.181
region [North Central]	−0.91	−1.81 – −0.01	0.048
region [Northeast]	0.30	−0.56–1.17	0.490
region [Pacific Northwest]	0.32	−0.48–1.13	0.430
region [Pacific Southwest]	0.01	−0.84–0.86	0.982
region [South Central]	0.40	−0.58–1.39	0.421
region [Southeast]	0.61	−0.29–1.50	0.185
month [2]	0.10	0.01–0.18	0.032
month [3]	0.10	0.01–0.19	0.032
month [4]	0.17	0.06–0.28	0.003
month [5]	0.24	0.10–0.38	0.001
month [6]	0.25	0.08–0.42	0.003
month [7]	0.30	0.11–0.49	0.002
month [8]	0.30	0.11–0.49	0.002
month [9]	0.27	0.10–0.43	0.002
month [10]	0.24	0.11–0.37	< 0.001
month [11]	0.12	0.02–0.22	0.016
month [12]	0.08	−0.02–0.17	0.108
year [2019]	−0.04	−0.09–0.00	0.079
agency [FWS]	−0.87	−1.56 – −0.17	0.015
agency [USFS]	−0.57	−1.05 – −0.09	0.020
Random effects			
σ^2^	0.13		
τ_00 siteid_	0.49		
ICC	0.79		
N _siteid_	97		
Observations	1558		
Marginal R^2^/Conditional R^2^	0.76/0.95		

### Model performance

**Fig. 3:**
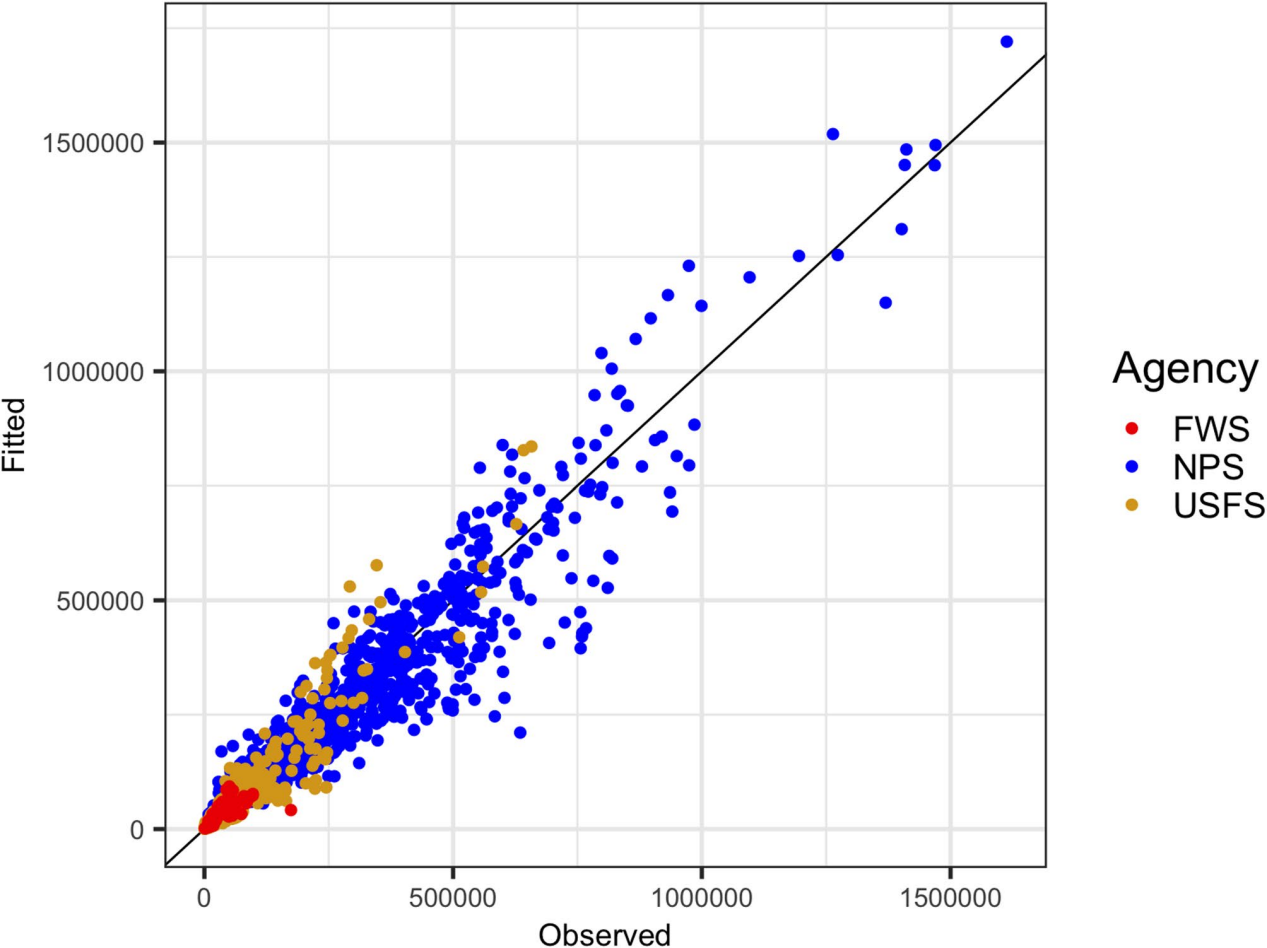
Scatter plot of regression model fitted visitation against the observed visitation in terms of monthly visits, colored by agency, showing the range of visitation this model spans and the range of visitation at units managed by each agency. Statistics from models for each agency fit separately can be found in the Supplementary Materials.

When controlling for agency differences, along with the other predictors, the base model fit well with a marginal R^2^ = 0.76 (variation explained by covariates alone) and conditional R^2^ = 0.95 (variation explained with covariates and the random effects) (Table 1; Fig. 3)^28^. All coefficients for the mobility data sources are significantly different from zero at the alpha = 0.05 level. The monthly coefficients show a consistent seasonality that is not explained by the mobility data and covariates alone. The negative coefficients on the agency dummy variables imply that, for the same level of other covariates and visitation mobility data sources, the observed monthly visitation for the USFS and USFWS units is generally less than for the NPS, the reference agency.

### Relative importance of predictor variables

**Fig. 4:**
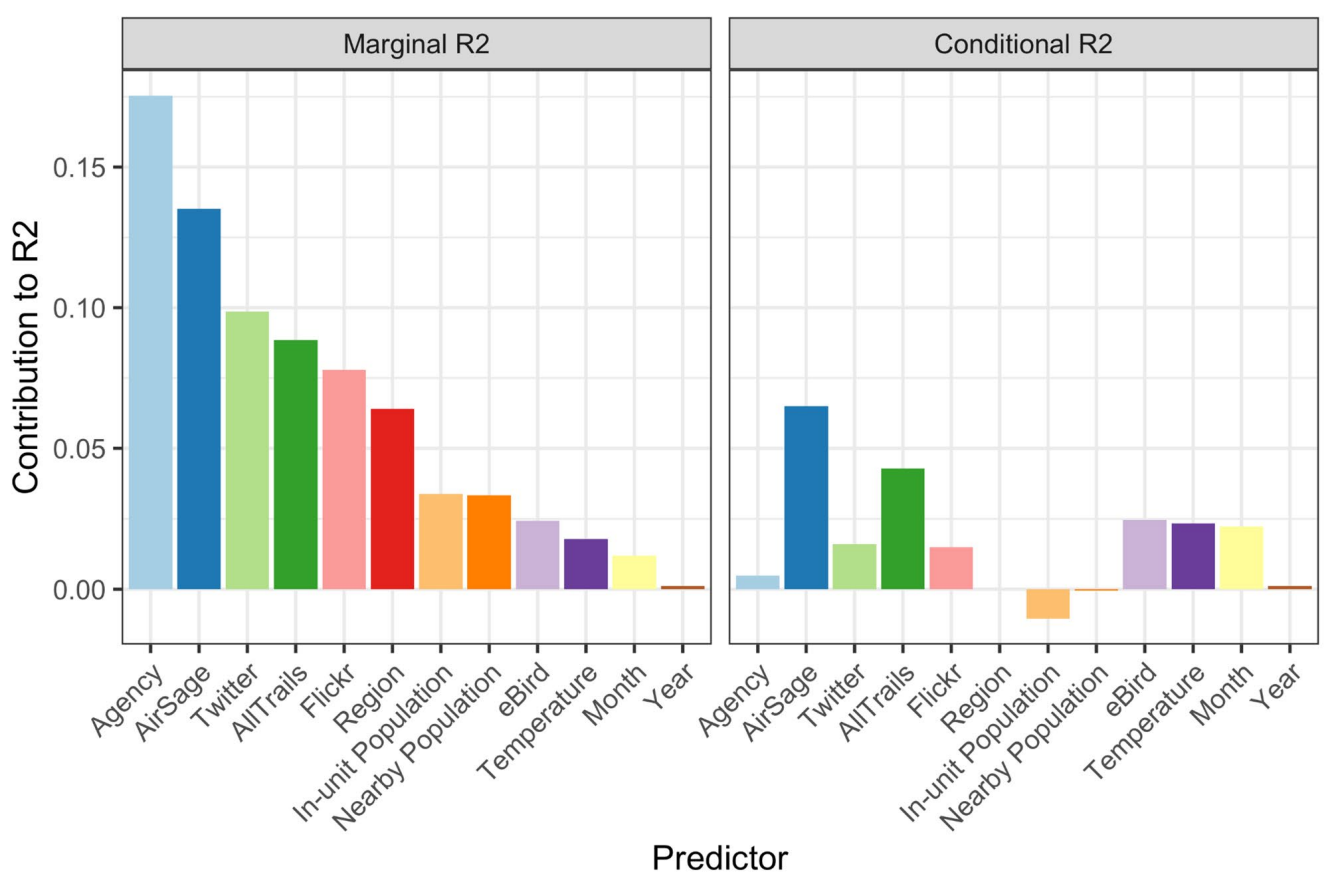
Relative importance plots for both marginal and conditional R^2^ statistics from the base model.

For marginal R^2^, knowing which agency the visitation series is from was the most valuable information (Fig. 4). Secondary to agency were the counts from the mobility data sources with the AirSage mobile device counts being the most useful followed by Twitter, AllTrails, and Flickr. The conditional R^2^–which provides similar results but assumes the random effect for each unit can be estimated using some on-site visitation data–shows AirSage, AllTrails, and eBird, followed by temperature and month as the most useful in explaining the remaining temporal variation.

### Cross validation for potential applications of the models

#### Across-agency transfer

**Fig. 5:**
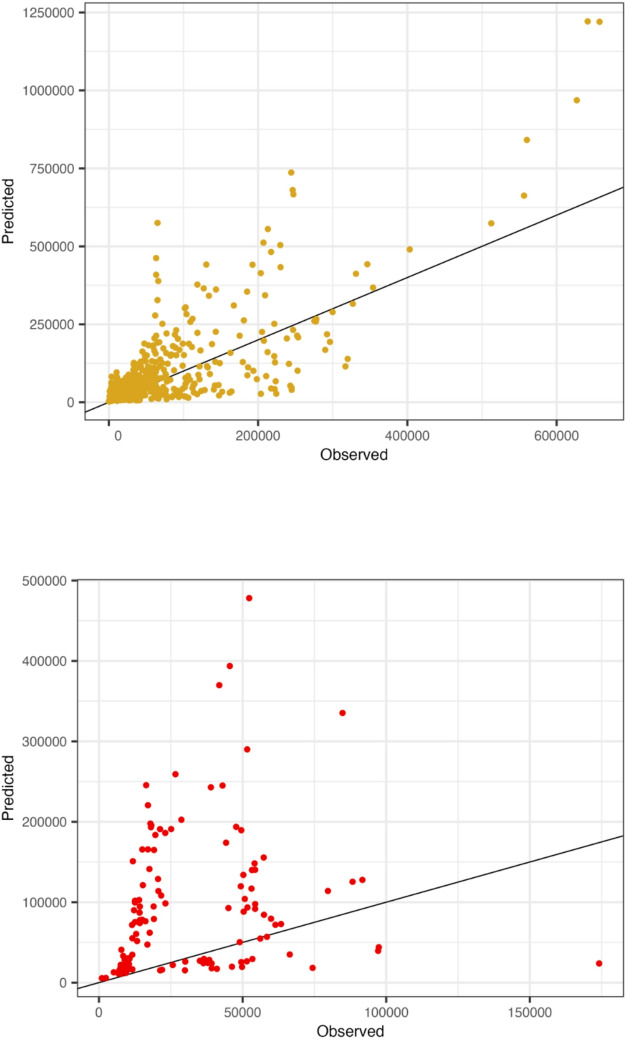
Scatter plots of the predicted against the observed values in terms of monthly visits for the USFS units (**a**) and the USFWS units (**b**) for predictive models fit on data from NPS units.

Agency is the most important covariate in explaining monthly variation in visitation across units (Fig. 4), implying that a model fit solely on one agency’s lands may not transfer well to those of another agency. We tested this further by creating a model fit on the NPS data and testing its performance when used to predict visitation at units managed by the other agencies. We see the models are biased and inaccurate in reproducing the new agency’s visitation data, with predictions for the USFWS worse than for the USFS (Fig. 5). Full cross-validation results using model variations including different combinations of mobility data and covariates are in the Supplementary Materials. However, none of the various models fit on the NPS are particularly successful at estimating visitation to USFS or USFWS units.

#### Across-units transfer

**Table 2:**
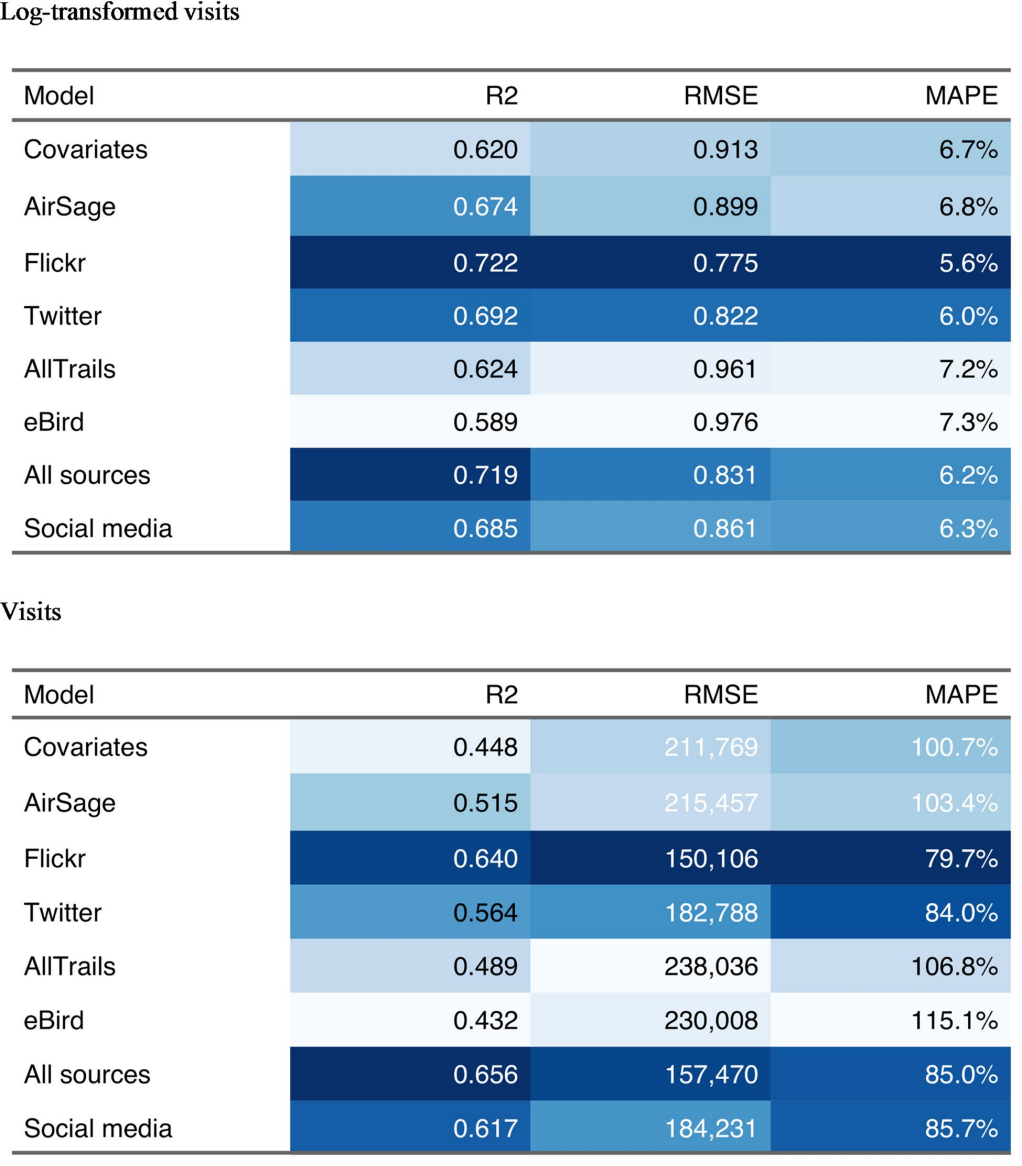
Cross-validation results for models holding out entire units. Columns are shaded from best (dark blue) to worst (light blue) according to each out-of-sample metric. High values of R^2^ are considered best, while low values of RMSE (root mean square error) and MAPE (mean absolute percentage error) are considered best. Results are presented in terms of both log-transformed and untransformed visits.

When models are estimated using on-site visitation data and digital mobility data from only a subset of units and then applied to other units using only digital mobility data, the predictions are generally inaccurate, with MAPE ranging from 80% to 115% in visit terms (Table 2-visits). These values represent average over- or under-predictions of monthly visits ranging from 150,000 to nearly 250,000 depending on the model specification. These results point to the difficulty of transferring a model estimated in one place to another place, with a key problem being potential over-fitting issues that are obscured when evaluating only the in-sample regression results presented earlier. This also implies using simple model performance measures derived from in-sample tests may lead to a false sense of accuracy in prediction of visitation at unstudied sites.

Looking across the possible model specifications, we find that the All sources model with mobile device location data included was only slightly better than those with only social media data. Models based on Flickr outperform the combined models in most dimensions, like R^2^, RMSE, MAPE in log and visit terms (Table 2), suggesting that this dataset continues to be useful in visitor estimation, despite its declining popularity.

#### Across-time transfer

**Table 3:**
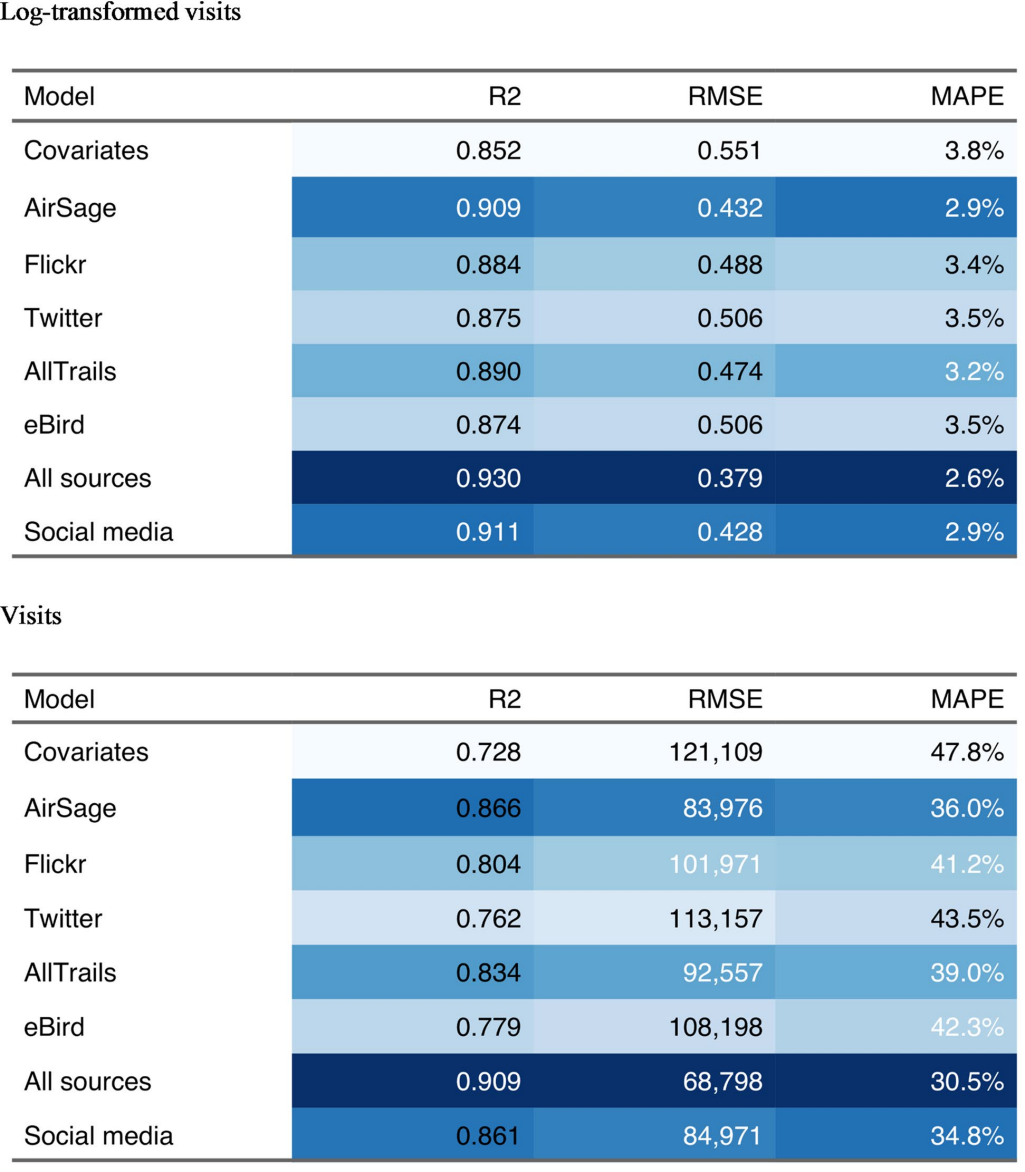
Cross-validation results for models holding out months. Columns are shaded from best (dark blue) to worst (light blue) according to each out-of-sample metric. High values of R^2^ are considered best, while low values of RMSE, and MAPE are considered best. Results are presented in terms of both log-transformed and untransformed visits.

When models are estimated using on-site visitation data and digital mobility data from all units for a specific time period and then applied to those same units during other time periods, using digital mobility data as predictors, the estimates are generally accurate and much more accurate than estimates of models transferred across units (Table 3). This higher model accuracy results primarily because differences across units can be accounted for with the random effect, since all units had at least some on-the-ground count data used in model estimation. This improvement can be seen with a lower MAPE of 31% in visit terms for the All sources model. This model using all mobility data sources as predictor variables performed better by all measures. The Social media model, using all social media data sources as predictors, was only slightly worse in comparison with the All sources model containing mobile device location data. Of the models making predictions based on individual mobility sources alone, the AirSage model using mobile device location data performed best, followed closely by a model dependent on AllTrails alone (Table 3).

## Discussion

Being able to measure public land visitation over time, across diverse landscapes, remotely and continuously, would revolutionize our understanding of the human use of, and impacts on, natural areas. Visitation models integrating digital mobility data on people’s movement and choices move this lofty goal closer to being operationally feasible^17,18^. As researchers in multiple fields of natural and social sciences, along with natural resource managers, start using this source of information, visitation data series and other inferences created based on such models must be critically assessed in terms of bias and accuracy for common intended uses. Misestimating visitation could lead to false signals and over-confidence in the derived data series for important uses, such as in natural resource damage assessment cases^4^ or in official reporting to decision makers who are weighing the expenditure of public resources^2^. A critical assessment, such as the one presented here, not only informs appropriate uses of models that integrate digital mobility data but also provides direction for future research and development.

Consistent with other studies^20,26^, we found that digital mobility data are generally well-related to observed visitation patterns for public lands. However, we showed this relationship can be quite poor for some individual units even when agency-wide correlations are good. Relationships varied considerably across different types of digital mobility data, units, and agencies. This is in keeping with previous work, which has found high site-to-site variability in the relationship between mobility data sources and on-site visitation, as well as model performance^18,20,26^.

Predictive performance of visitation models varied widely across sites but was improved with the inclusion of some directly observed visitor count data from the units of interest. To see the practical implications for visitation estimation, we compared the accuracy of visitation models with and without on-the-ground data from the units of interest included in the construction of the models. The best-performing model in the across-time transfer (that includes some on-site data from each site) predicted monthly visits with a MAPE of 30.5% (in untransformed visit terms). That same All sources model had an 85% MAPE when applied to predict visits at new locations (without on-site data), which is substantially worse. These results highlight the challenges of building models of visitation across diverse geographies and unit types, while building evidence for the currently most-promising uses given the present limitations. These promising uses of visitation models driven by mobility data might include imputation of missing values, real-time forecasting, or complementing on-the-ground methods in a hybrid visitation modeling approach. While we did not explore it here, understanding how much on-the-ground data may be needed for a given level of desired accuracy in a hybrid approach is a natural direction of future research.

We found that mobility-data-based models fit on one agency’s visitation data were generally poor and biased predictors of another agency’s observed visitation. The differences we found across agencies points to differences in how the mobility data sources represent use across these agencies’ lands. It may also point out fundamental biases in the agencies’ count systems and statistical methods to create monthly visitation totals. In other words, they may not be counting and reporting in a way that creates comparable metrics on the same visitation scale^29^. The same logic holds for the differences we found between units within an agency. The models we created cannot tell us where the differences are coming from, but future studies are warranted on that topic.

While we quantitatively assessed the bias and accuracy of the visitation models using mobility data, information for assessing the accuracy of established methods of visitor-use quantification and resultant data series is rare^30^. Knowing which on-the-ground series are more reliable and unbiased could improve our ability to disentangle the differences we found across agencies and units in our models. There is a need to harmonize and standardize the on-the-ground visitation series creation and reporting across a diverse set of natural lands (e.g., parks, beaches, and forests). One potential direction for research aimed at reducing the deviations is to better match the spatial definitions of the areas of interest for the mobility data (i.e., the geofence) and the areas that the traditional counting methods are capturing. This spatial definition is not always clear for on-the-ground visitation data series and similarly the choice of geofence can cause large discrepancies in mobility datasets if it mistakenly captures the use of nearby high-use infrastructure such as roads. Without more formally documented and widely available visitation observations, general predictive models built on mobility data will struggle to improve in usefulness. This means there is a need to potentially conduct more, not less, traditional visitation quantification methods to improve the novel approaches.

The potential for commercial mobile device location data as a mobility data source to fill a wide array of research and management needs has been touted in peer-reviewed literature^17,31^, popular media^32^, and legislation^14^. The quantity of mobile device location data, its spatial resolution, and near real-time availability are often assumed to be advantages for modeling visitation, compared with other forms of digital mobility data. While we did find mobile device location data to be the most useful individual digital mobility data source for explaining variability in visitation in most cases, it was not substantially so. Our models that incorporated multiple digital mobility data sources were still more accurate than models based solely on mobile device location data, or any other individual source. Additionally, the marginal contribution of adding the mobile device data to a model already using a set of combined social media sources was small. Our findings provide some evidence that if researchers or managers can only incorporate one type of digital mobility data in their statistical visitation model, mobile location data may be the best choice.

It is important to note that there are many commercial mobile device location data vendors with varying location data panels and processing pipelines available, and we only evaluated one of these (AirSage). A more detailed description of this vendor’s data and processing is described by Tsai et al.^26^, who highlight how the proprietary and opaque data pipelines and processes used by mobile location data vendors makes rigorous scientific scrutiny of the sample characteristics and stability difficult. More research is needed to confirm that our findings hold across different vendors, settings, and in future years given continuing changes in the input data from mobile devices and the algorithms used by the vendors of location data. One recent study^20^ observed that vendors’ datasets differ in explanatory power, not only across vendors but across products provided by the same vendor. There are clear needs for more transparent and explainable sources of location data if they are to be more-widely integrated into visitor use monitoring programs^10^, which will depend on development of models that account for biases in representativeness of the mobility data.

We did not address temporal stability of the models in this study, as we were only able to combine two years of overlapping data across all geographies. While we did not find significant year effects between those two years, the mobility data sources are undeniably changing over time with differences in smartphone application popularity, privacy settings, and use, resulting in changes in the panel makeups in the mobile device products^33,34^. These instabilities have implications for predictive models, as the temporal changes in mobility data sources have proven differences in relationships with on-site visitation estimates^20^. Future research is needed to quantify the impact of temporal instability on predictive performance of models using mobility data sources and create and test approaches that combine on-the-ground counts with mobility data to handle the changes in practical applications. As social media and other locational data sources change (new datasets become available and others lost) predictive models will need to be adjusted. This underscores the ongoing need for reliable and accurate on-the-ground visitation series for ongoing calibration and validation of visitation models based on digital proxies.

The commercial mobile data performed similarly to the individual social media sources and evidence suggests it should be treated as a promising member of a group of potential visitation predictors, but it does not fully bridge the more challenging gap in our understanding of differences in visitation across units. Combining multiple sources of digital mobility data improved the models compared to those based on individual sources. This finding is not always true, however. For example, we found evidence that activity-specific mobility sources, such as AllTrails, may perform well in settings where the captured activity (predominantly hiking) represents a good portion of the variation in visitation to those locations (e.g., USFS). As the sources of available mobility data evolve over time, there may be a place for ensemble modeling systems with common ground-collected benchmarks^35^.

Given the potential expense and complications of maintaining a model or set of models with multiple, potentially changing data streams as inputs, a productive direction of future research is in constructing and testing more streamlined and benchmarked models. In that direction, an assessment of acceptable bias and accuracy from land managers could inform what the range of “good-enough” may be for real-world applications. We tested the use of location data inputs separately and in combinations by using cross validation routines that penalized over-fitting and matched potential model transfers with likely use cases, as opposed to only presenting in-sample fit statistics that would give an over-optimistic representation of the expected predictive performance. We collected a set of covariates that were known to be useful predictors of visitation from previous work. However, there may be other predictors that could produce reasonably accurate, yet parsimonious, models of visitation for specific or groups of public land units. Assessment routines, such as the ones presented here, could provide a common set of measuring techniques to decide which specific model constructions and covariate combination meet the needs of simplicity and practicality in operation, and bias and accuracy in prediction.

## Methods

### Study area and timeframe

**Fig. 6:**
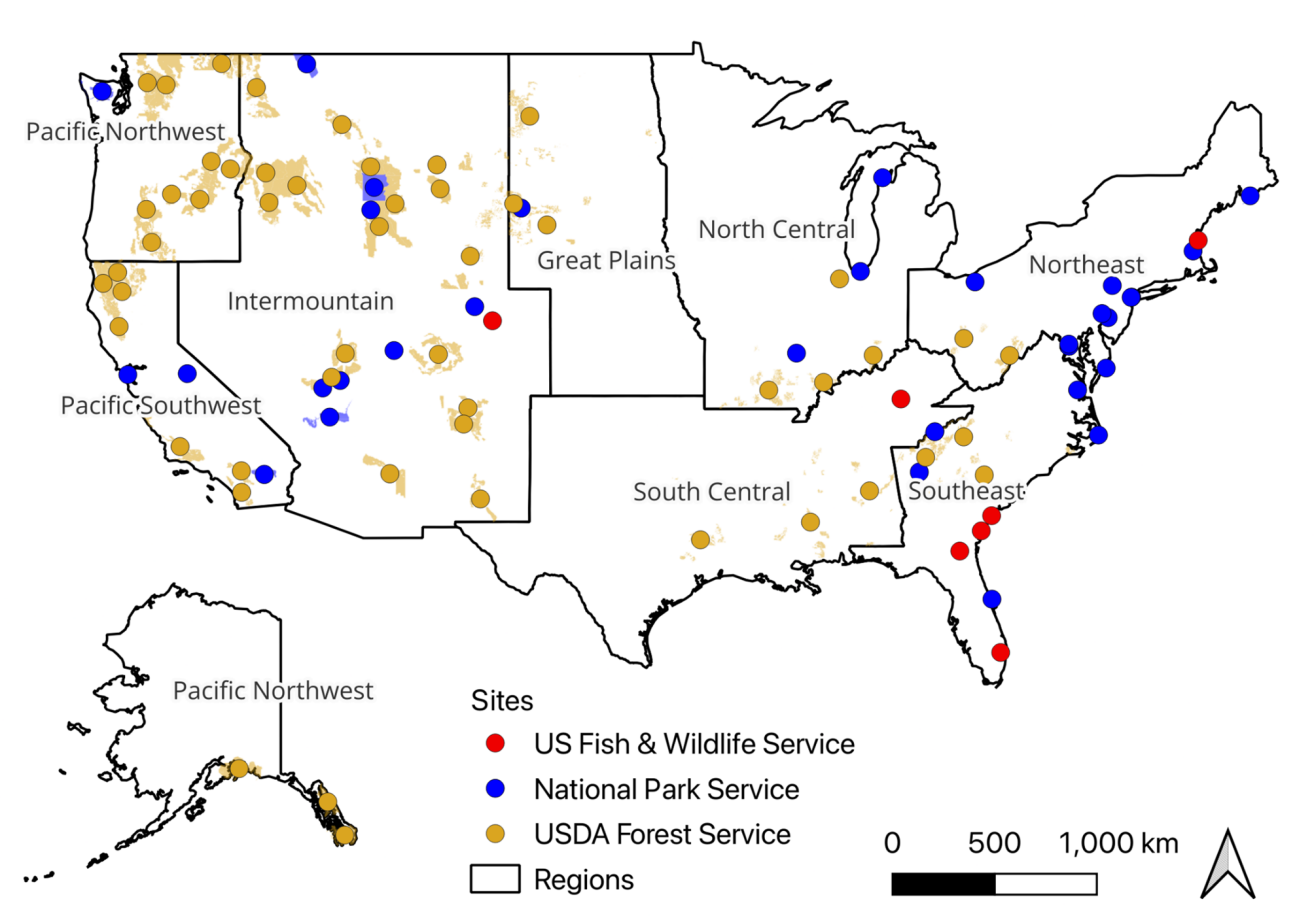
Map of study locations and regions, colored by management agency. Shaded areas show unit boundaries for units which are larger than the points.

We studied public lands managed by the U.S. National Park Service (NPS), Forest Service (USFS), and Fish and Wildlife Service (USFWS), during 2018 and 2019 (Fig. 6). The NPS units were 38 of the 50 most visited units in 2018 and 2019, excluding NPS parkways and units with road network issues, special events (e.g., large festivals) that altered recreation characteristics, and extended closures during the study years (as in^26^). We included the 49 USFS units sampled under the USFS National Visitor Use Monitoring (NVUM) Program in federal fiscal years 2018 and 2019, and three USFS units sampled in federal fiscal year 2020 (to cover calendar years 2018 and 2019). Finally, we included seven USFWS national wildlife refuges with on-the-ground visitation data collected by refuge staff.

To obtain the digital mobility data, we first defined the geographic areas of interest. For the NPS units, we used the administrative boundaries from the DataStore at the NPS website (https://irma.nps.gov/DataStore/, as used in^26^. We used the administrative boundaries of the USFS units (https://data.fs.usda.gov/geodata/edw/edw_resources/meta/S_USA.AdministrativeForest.xml*)* to define the polygons for the mobile device location data. These administrative boundaries included private inholdings surrounded fully by USFS land and sometimes populated areas immediately adjacent to USFS lands. We used a more refined layer of USFS land ownership (https://data.fs.usda.gov/geodata/edw/edw_resources/meta/S_USA.BasicOwnership.xml*)* to define the USFS polygons of interest for which social media data was obtained. We used the FWS National Realty Boundaries from the FWS Cadastral Database (https://ecos.fws.gov/ServCat/Reference/Profile/121225*)* to define the FWS polygons.

### Mobile device location and social media data

We sourced a commercial mobile device location-derived visitation dataset from AirSage Inc., which provided aggregated estimates of population-level, daily visitation during 2018 and 2019 to our defined points of interest (geographic units described above) based on their panel of mobile device locations and their proprietary processes. These daily estimates were summed to generate monthly visit estimates. For the NPS, the AirSage data is what was used by^26^. For the USFS and USFWS, we purchased equivalent retrospective AirSage data for our study period.

For every geographic unit we gathered data from three geolocated social media platforms (AllTrails, Flickr, and Twitter) and the geolocated community science platform eBird. Flickr images were retrieved by querying the Flickr application programming interface (API) in January 2023. Twitter posts were downloaded in real-time from Twitter’s “statuses/filter” streaming API between January 2018 and December 2022 (before the platform was renamed X). eBird checklists^36^ were requested from the eBird administrators and received in January 2023. We used a spatial join to identify all AllTrails locations (downloaded from the AllTrails website in January 2023) within the boundaries of the units, then counted the number of trip reports for each location based on information displayed on the AllTrails website in January 2023 (following^37^.

Following the collection of the social media data, we quantified the numbers of users each day who shared posts on each social media and community science platform who were present within the boundaries of each unit. Then, for each data source, we calculated the number of user-days (unique users in a given location on a given day^21^;. for each unit and summed these into monthly totals of user-days per data source per unit.

### Unit visitation data

Monthly park visitation to NPS units was retrieved from the NPS Stats database (https://irma.nps.gov/STATS/*)* recreational visit series and are those described by^26^. For USFS units, monthly visitation estimates were created with data from the NVUM Program, which generates unit-level recreation estimates that cover a 12-month period corresponding to U.S. federal fiscal years (October through September)^38^. Each unit receives a visitation estimate once every five federal fiscal years. We converted these annual estimates into monthly figures by adapting the “all-forest information” downscaling approach for NVUM data described by^37^. Because the federal fiscal year spans two calendar years and a subset of USFS units are sampled in each fiscal year, we were able to use USFS units representing a diverse array of types and settings to cover the 24 months of our study. In total, our analysis included data from 52 forests and the number of forests represented in any single month was 25 (Jan 2018 - Sept 2018), 24 (Oct 2018 - Sept 2019), and three (Oct 2019 - Dec 2019). For USFWS wildlife refuges we acquired data on refuge visits from individual refuge managers whom we contacted as part of a related project and are those described by^20^. We vetted the methods reported by the refuge managers and selected refuges with robust visitor estimation techniques.

### Covariates

We collected six covariates that have been found to be useful for visitation modeling in past studies. A correlation matrix for the covariates can be found in the Supplementary Materials (Figure A1).

#### Region

To capture variation in visitation due to differences in recreational behavior across the U.S., we assigned each unit to one of eight regions (Pacific Northwest, Pacific Southwest, Inter-Mountain, Great Plains, North Central, South Central, Northeast, and Southeast; Fig. 6). These regions are based on the subregions developed for the USFS Resources Planning Act Assessment which capture broad ecological and socioeconomic patterns across the U.S. For the assignment, we intersected a shapefile of the regional boundaries with the centroids of each unit. Eight unit centroids were located in oceanic waters where the regions are not defined and so were assigned to the nearest region manually.

#### Population within unit boundaries

We defined the AirSage data of interest using the administrative boundaries for the USFS forests; these boundaries include some privately-owned lands within (i.e., inholdings surrounded by USFS land) and on the periphery of USFS land that can contain populated areas. To control for the AirSage data that could be attributed to the daily activities of people living within the administrative boundary that are unrelated to recreating on USFS land, we estimated the population living within the unit boundaries (administrative boundaries for the USFS, standard unit boundaries for the NPS and FWS). To do this, we intersected a shapefile of the unit boundaries with a rasterized map of estimated population at a 30 m cell resolution^39,40^, then summed the population estimates for each intersected cell within a unit.

##### Population within 30 Miles

We hypothesized that the number of people living near a unit would have an effect on visitation, with more people providing a larger pool of potential local visitors. To quantify the impact of nearby population, we estimated the population living within a 30-mile straight-line distance of the unit boundaries (ownership boundaries for the USFS, standard unit boundaries for the NPS and FWS). We chose 30 miles as the distance from the park boundaries based on previous research from the USFS that defined local visitors as those travelling 60 miles or less to the recreation site and found that a travel distance of between 50 and 60 miles corresponded with 30 straight-line miles from the forest boundary^38^. More than half of all visits annual to Forest Service lands are associated with people living within this 30-mile distance. We intersected the 30-m resolution population raster with the geometries of the units after applying a 30-mile buffer, and summed the population estimates for all cells that intersected the buffered boundaries.

##### Temperature

To capture the effect of climate and weather on visitation, we included temperature in the model. We used mean monthly temperature from the *ERA5-Land datase*t which is provided at a 0.1 degree latitude by 0.1 degree longitude resolution. We intersected the geometries of the units with the centroids of the rasterized temperature map, then averaged across all intersected cells within a unit by month. Some units were small enough to not intersect with any temperature cell centroid, in which case we used the data from the cell with the closest centroid to the unit.

##### Month and year

To capture seasonality in recreation behavior we included categorical variables indicating the year (2018 or 2019) and the month of the year.

The datasets and code to reproduce the results can be found here: https://github.com/OutdoorRD/national-model-paper.

### Correlation among variables

We calculated Pearson’s correlation coefficients between each visitation source and the observed visitation records overall and for each agency. Within each agency, we also calculated correlations between each visitation mobility data source and the observed visitation by unit and by year, to understand the variability in correlation across time and space.

### Relative contributions of mobility data

We used linear regression and a dominance analysis to address RQ1, which asked whether different mobility data sources vary in their contribution to variance explained by a visitation model. Our model was a random effects mixed model that explained variation in monthly visitation across all units and agencies:

1 $$\:log({{y}_{it})=\alpha\:+{\beta\:}_{1}{log(C}_{i})+{\beta\:}_{2}{T}_{it}+{\beta\:}_{3}log\left({P}_{it}\right)+{u}_{i}+{e}_{it}\:}_{}$$

where:

$\:{y}_{it}$ - Monthly visitation to unit *i* in month *t*.

$$\:\alpha\:$$ - global intercept.

$$\:{\beta\:}_{}$$- coefficient vectors.

$\:{C}_{i}$ - time invariant covariates (agency, population, region) for unit *i*.

$\:{T}_{it}$- time-variant covariates (temperature and calendar month) for unit *i* in month *t*.

$\:{P}_{it}$ - social media and mobile data visitation estimates for unit *i* in month *t*.

$\:{u}_{i}\:$- random site-level effect for unit *i*.

$\:{e}_{it}$- error term for unit *i* in month *t*.

We fit the regression models in R version 4.2.2^41^ and the lme4 package^42^ assuming $\:{u}_{i}\:$and$\:{\:e}_{it}$ are normally distributed. The random effects model requires strong assumptions that are often violated in practice, such as the random effects being uncorrelated with the included covariates, or else inducing bias^43^. However, a fixed effects specification for Eq. 1, would not have allowed us to predict visitation to the held-out test sets of units in the across-agency or across-units CV routines presented below (Table 4), nor would it allow us to estimate the effect of time invariant covariates. To check the sensitivity of our results to this assumption, we fit a fixed effects regression model as well. The results from this regression are presented in the Supplementary Materials (Table A7).

We conducted a dominance analysis to quantify and plot the marginal contribution of each predictor to model performance using the dominanceAnalysis package for R^44^. Dominance analysis is a class of techniques used to quantify the relative value of including individual covariates in a model, typically represented in terms of improved goodness-of-fit metrics. We quantified the increased R^2^ when each predictor was added to the model formulation, also known as a Shapley value decomposition^45^. It was calculated for models with every possible combination of predictors and then averaged across all formulations. Since our model is a mixed model with unit-level random effect groupings, we quantified the relative contribution of each variable to two different measures of model fit: marginal and conditional R^2^^28^.

### Model transferability across agencies, locations, times

We created three cross-validation (CV) routines to simulate potential applications for predictive visitation models to address RQ2 and RQ3 (Table 4). Each CV represents, sequentially, a conceivably “easier” task for model prediction. These tests are useful for understanding the visitation prediction accuracy of models in ways managers and researchers may want to apply them.

**Table 4:** Scenarios in which managers or researchers may want to apply visitation models, and the descriptions of the cross-validation routines we used to evaluate prediction accuracy of each. For each scenario, we performed the CV routine on eight distinct models representing four variations of inputs to the base model (Table 5).

Scenario	Training data	Tested on	Results presented
Across-agency transfer	NPS	USFS, USFWS	Predicted vs. observed plots, R^2^, RMSE, and MAPE on both log- & visit-scales
Across-units transfer	90% of units (across NPS, USFS, USFWS) in 10-fold CV	10% of units (across NPS, USFS, USFWS) in 10-fold CV	R^2^, RMSE, and MAPE on both log- & visit-scales
Across-time transfer	90% of unit-months (across NPS, USFS, USFWS) in 10-fold CV	10% of unit-months (across NPS, USFS, USFWS) in 10-fold CV	R^2^, RMSE, and MAPE on both log- & visit-scales

#### Across-agency transfer

One use may be to fit a visitation model on one agency’s data and use that to predict visitation to a different agency’s units. To simulate this, we fit our base model (Eq. 1) on the NPS observations then used that model to predict the visitation series for the USFS and USFWS. We opted to use the NPS as the initial agency because the NPS monitoring data is the most extensive within our analysis. This method simulates a potential use-case in which a practitioner transferred a model fit on one observational series to prediction for units managed by a different agency, which might lack as robust of a visitor monitoring program. We created scatter plots of observed vs. predicted values for each of the two predicted agencies (USFS and USFWS). We also calculated goodness-of-fit metrics including the coefficient of determination (R^2^, calculated by squaring Pearson’s correlation between predicted and observed values), root mean square error (RMSE), and mean absolute percentage error (MAPE).

#### Across-units transfer

Another use may be to predict visitation to unmonitored units in the same agency. We created visitation models using a subset of units and then used those models to predict visitation for the other units in the same time period. This application reflected the situation where an agency has an existing on-site visitation monitoring system that is implemented at some agency units, and a model could be used to predict visitation at unmonitored units. We simulate this use by creating a repeated k-fold cross validation where folds are whole units across all agencies. We used a 10-fold cross validation, repeating a random draw of an average of 9.7 units in each fold, 100 times, and created the same goodness-of-fit metrics as above based on the predictions on the held-out folds.

#### Across-time transfer

Lastly, visitation models may be used as part of a hybrid monitoring approach in which some on-site visitor counting is done in every unit, but only intermittently. This use-case is analogous to the USFS recreation monitoring system, where on-site monitoring happens every fifth year but there may be a desire to create visitation for unmonitored times, or a case of using a model to fill in months missing from visitation series due to staffing or equipment failure. We simulated this application by using the same 10-fold cross validation process described in the across-unit test, except here the folds are random sets of an average of 156 months across all units.

To judge the individual and combined mobility data sources’ efficacy for these three applications, we performed each CV routine on four variations of inputs to the base model (Eq. 1, Table 5):

1. Covariates - a model with just the covariates, not including mobility data sources.
2. Individual mobility data source- models with the standard covariates in Eq. 1 and one mobility data source, with each individual source in a separate model.
3. All sources - a model with all the standard covariates and all mobility sources together (our base model).
4. Social media - a model with all the covariates plus the social media mobility data.

For each variation we produced common fit metrics for both estimated visits and log-transformed visits. These CVs help us answer RQ2 and RQ3 for potential uses of visitation models, while the variation in the inputs to the models (1–4 above) help us answer RQ1 in a prediction setting as opposed to using in-sample results in the dominance analysis.

**Table 5:** Model variations which were tested in the CV routines (Table 4) to evaluate the relative contribution of each predictor to predictive performance. An “X” indicates that a predictor was included in a particular model variation. “Covariates” includes region, population within unit boundaries, population within 30 miles, temperature, month, and year.

Model Variations	Model Predictors					
	Covariates	AirSage	AllTrails	eBird	Flickr	Twitter
Covariates	X					
Individual mobility data sources						
AirSage	X	X				
AllTrails	X		X			
eBird	X			X		
Flickr	X				X	
Twitter	X					X
All sources	X	X	X	X	X	X
Social media	X		X	X	X	X

## Supplementary Information

Below is the link to the electronic supplementary material.

Supplementary Material 1

## Data Availability

Data and code are available at https://github.com/OutdoorRD/national-model-paper. Contact Nathaniel Merrill (nate@matunuckresearch.com) for any questions.
