# Supplementary material for: A national model for estimating United States public land visitation
Source: Sci Rep. 2025 Nov 28;15:42764. doi: 10.1038/s41598-025-26926-w (PMC12663154; doi:10.1038/s41598-025-26926-w)
Supplement: Supplementary file 1 — Supplementary Material 1 [file 41598_2025_26926_MOESM1_ESM.docx]

**Supplementary Materials
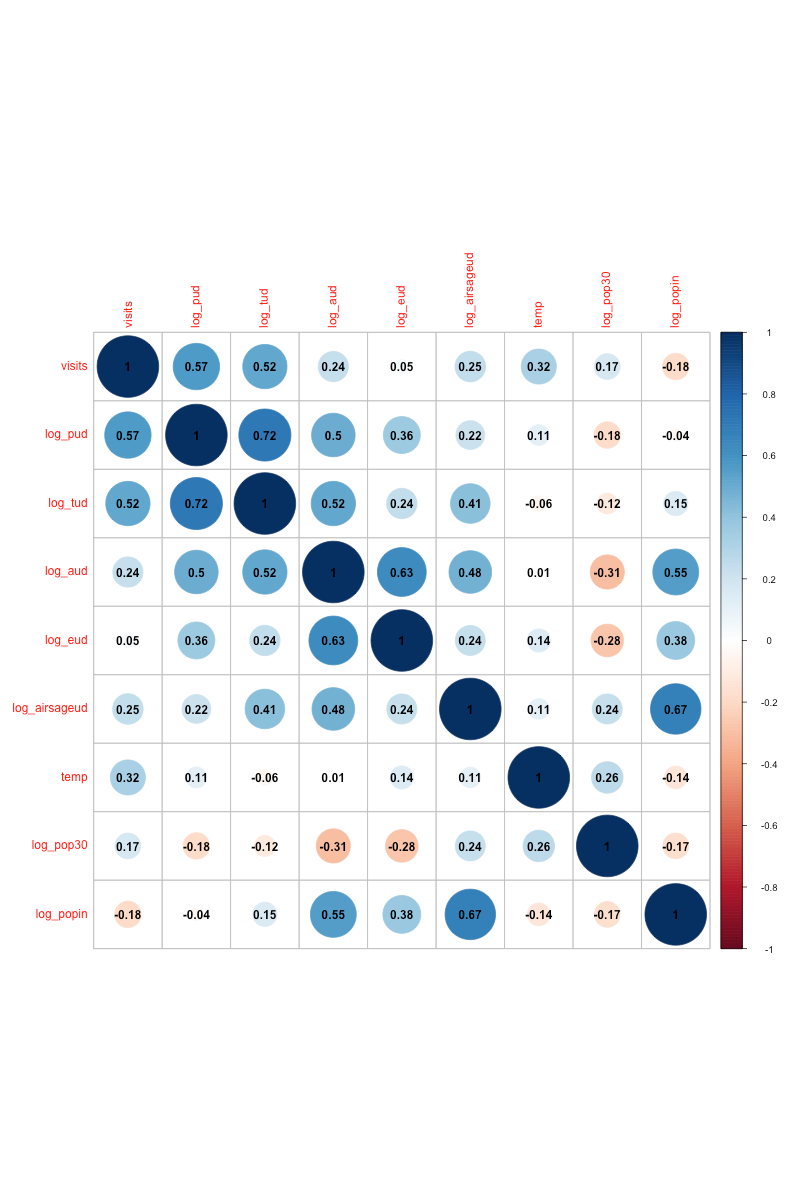
**

**Figure A1:** Pearson correlations between covariates in regression

**Table A1-** Regression for each agency separately


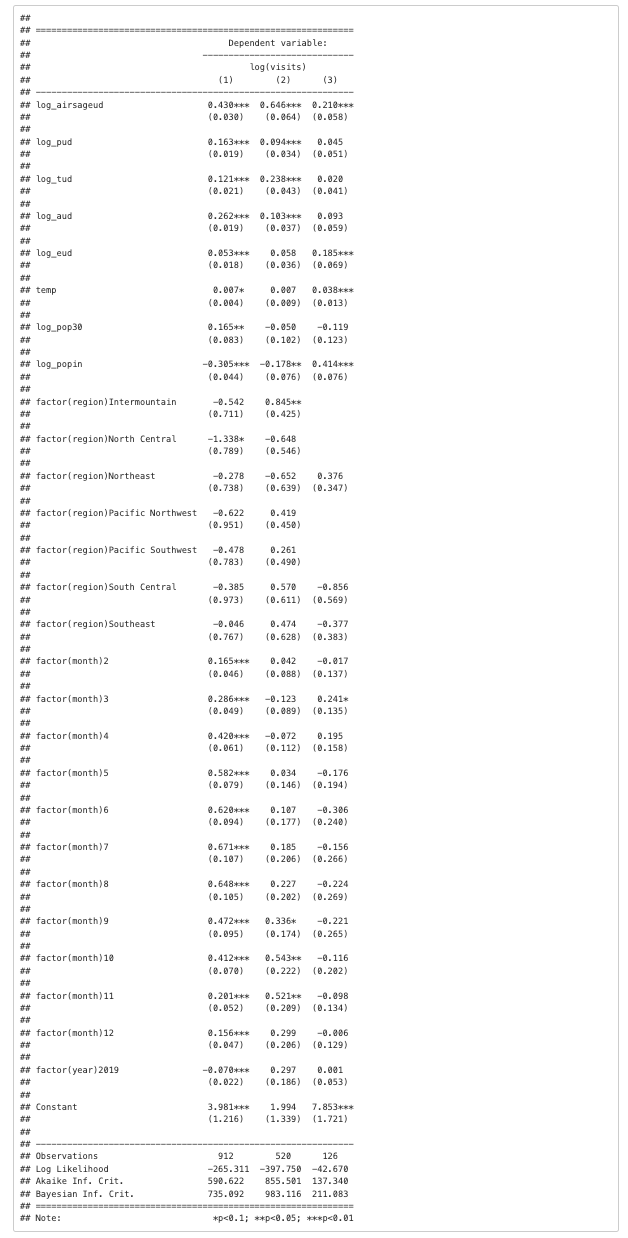


Note: regression (1) NPS, (2)USFS, (3)USFWS


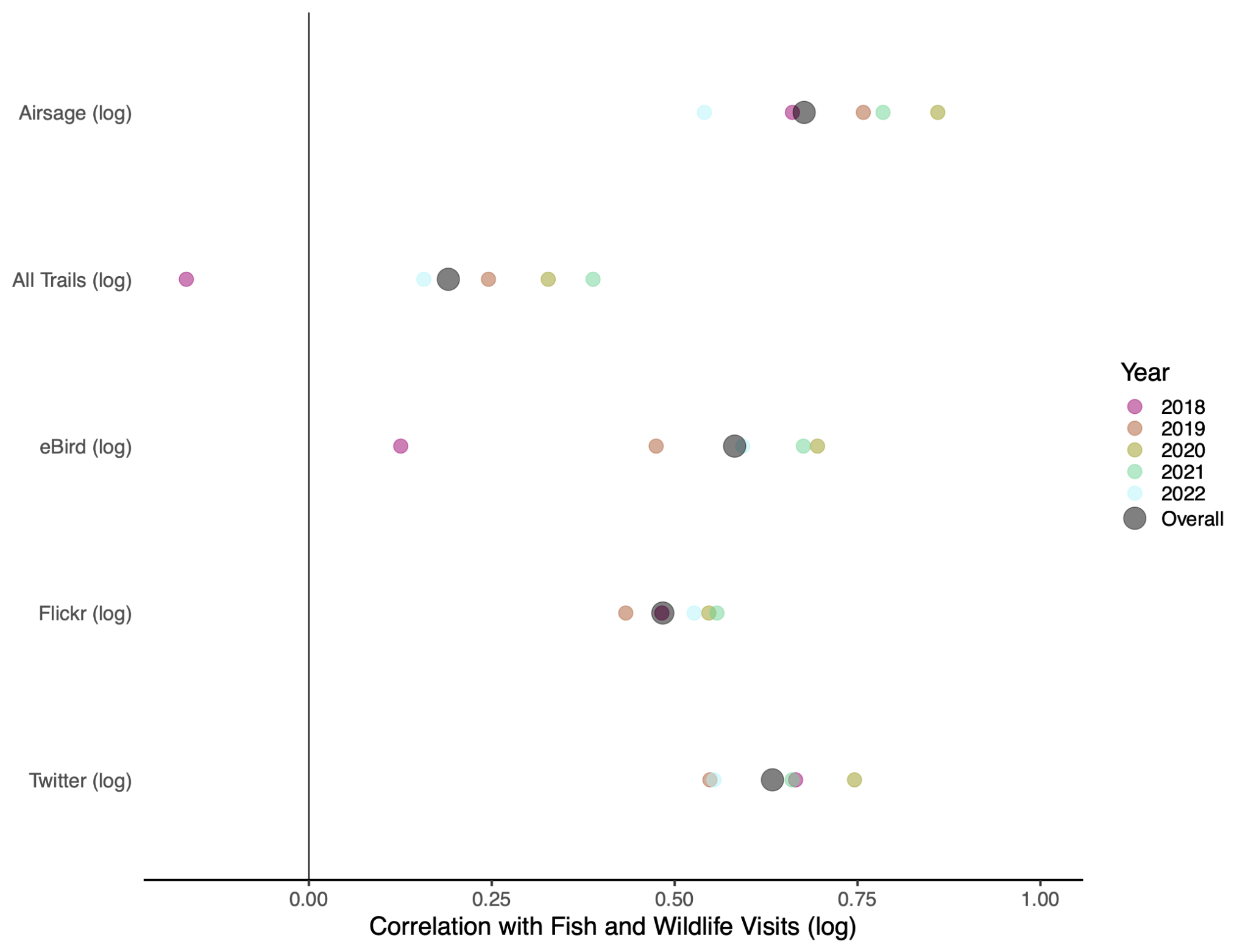


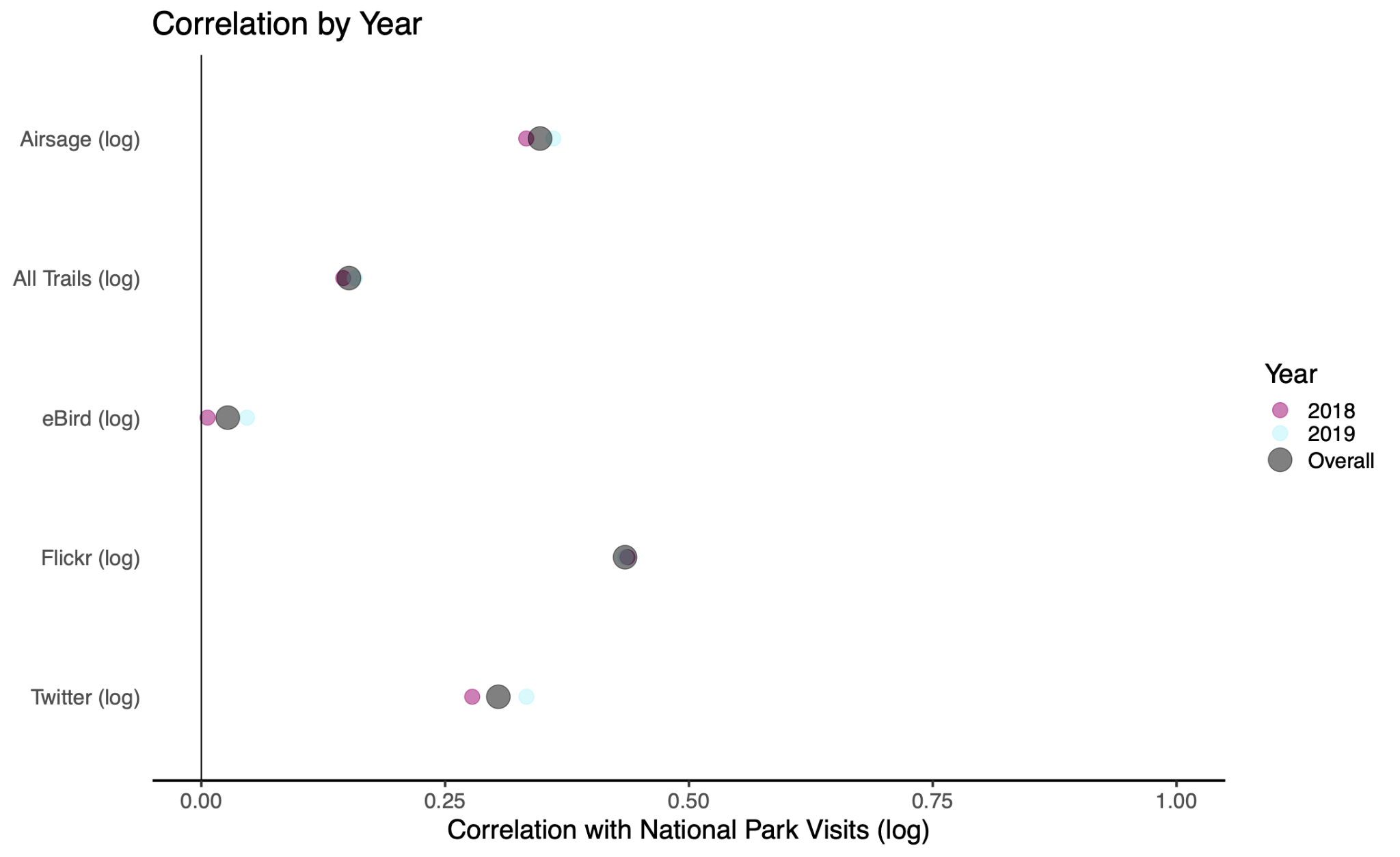


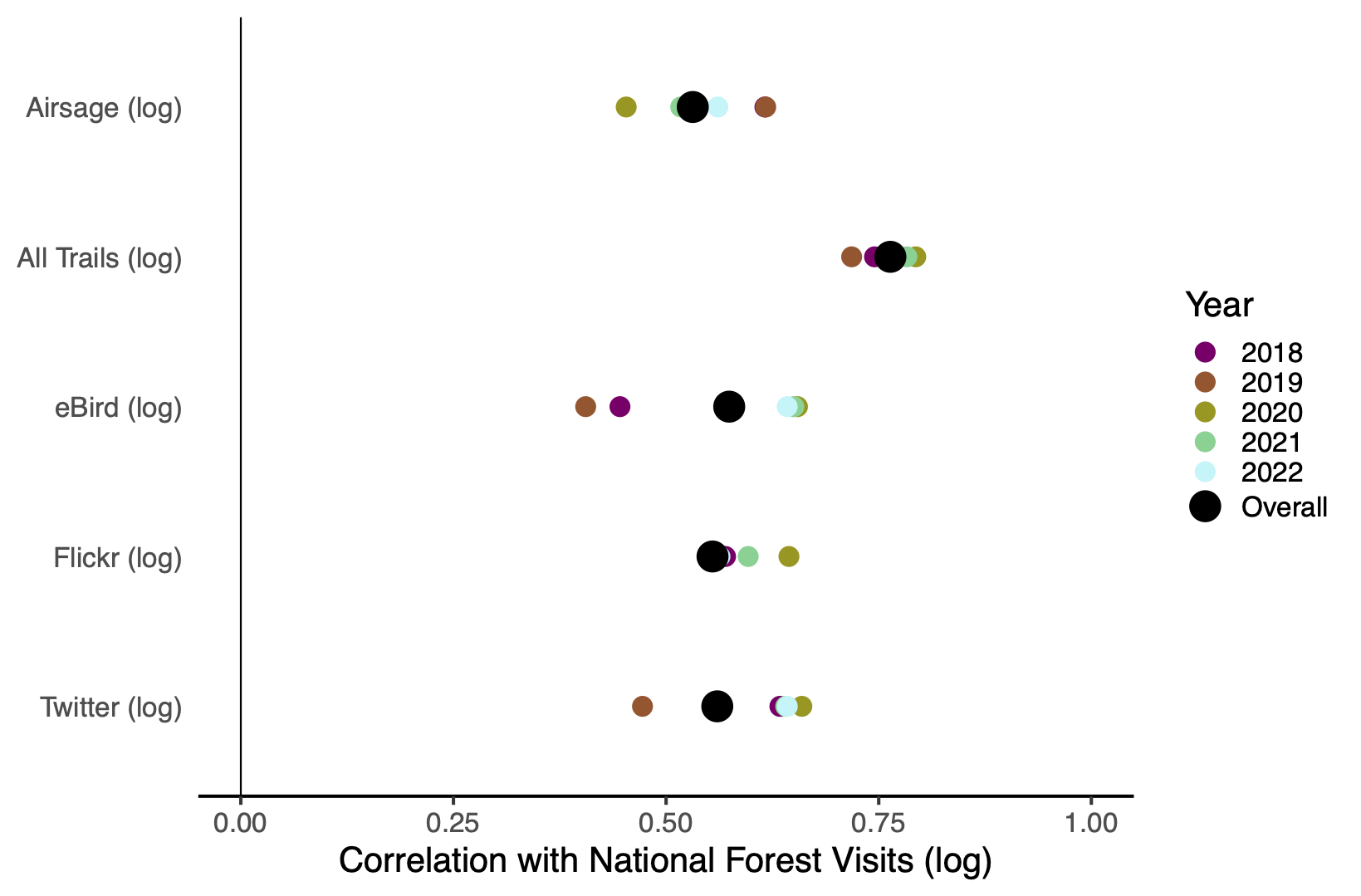


**Figures A2-4:** Correlations between mobility data sources and agency visitation (USFWS, NPS, USFS) by year

**Tables A2-5:** full CV results for models fit to NPS and transferred to USFS and USFWS

**Table A2**: Cross-validation results for the USFS based on models fit to the NPS.

logs


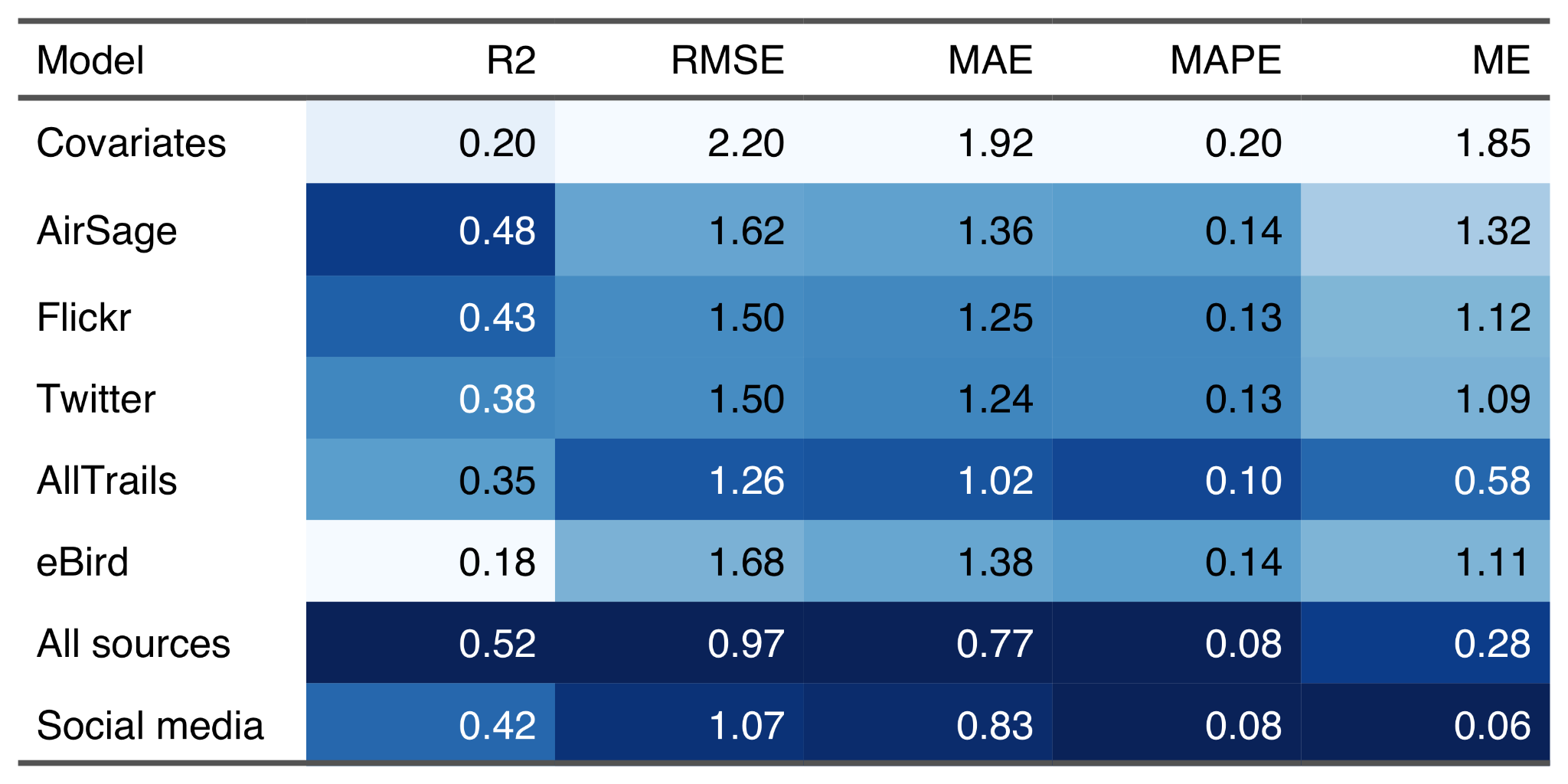


visits


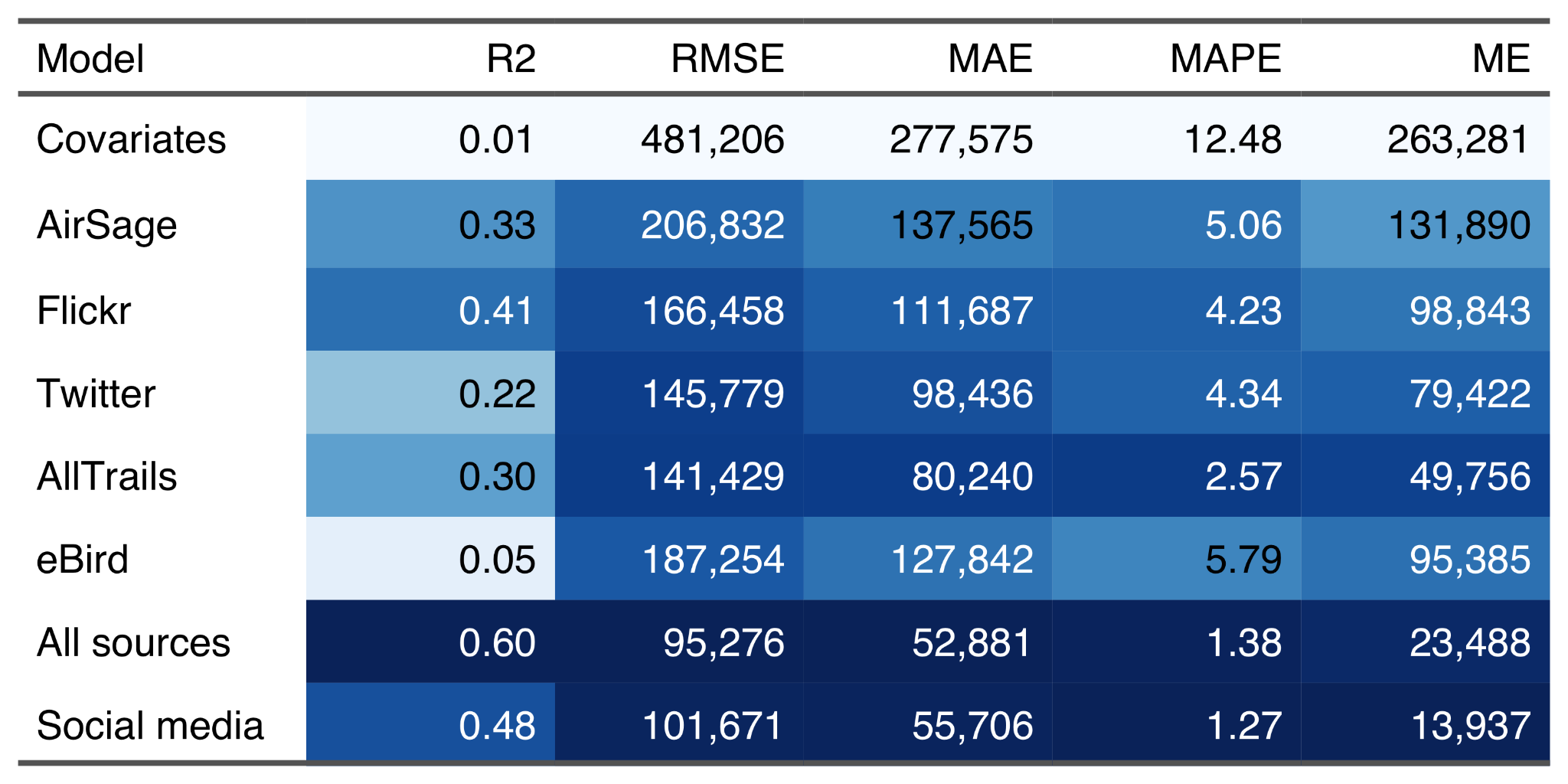


**Table A3**: Cross-validation results for the USFWS based on models fit to the NPS.

logs


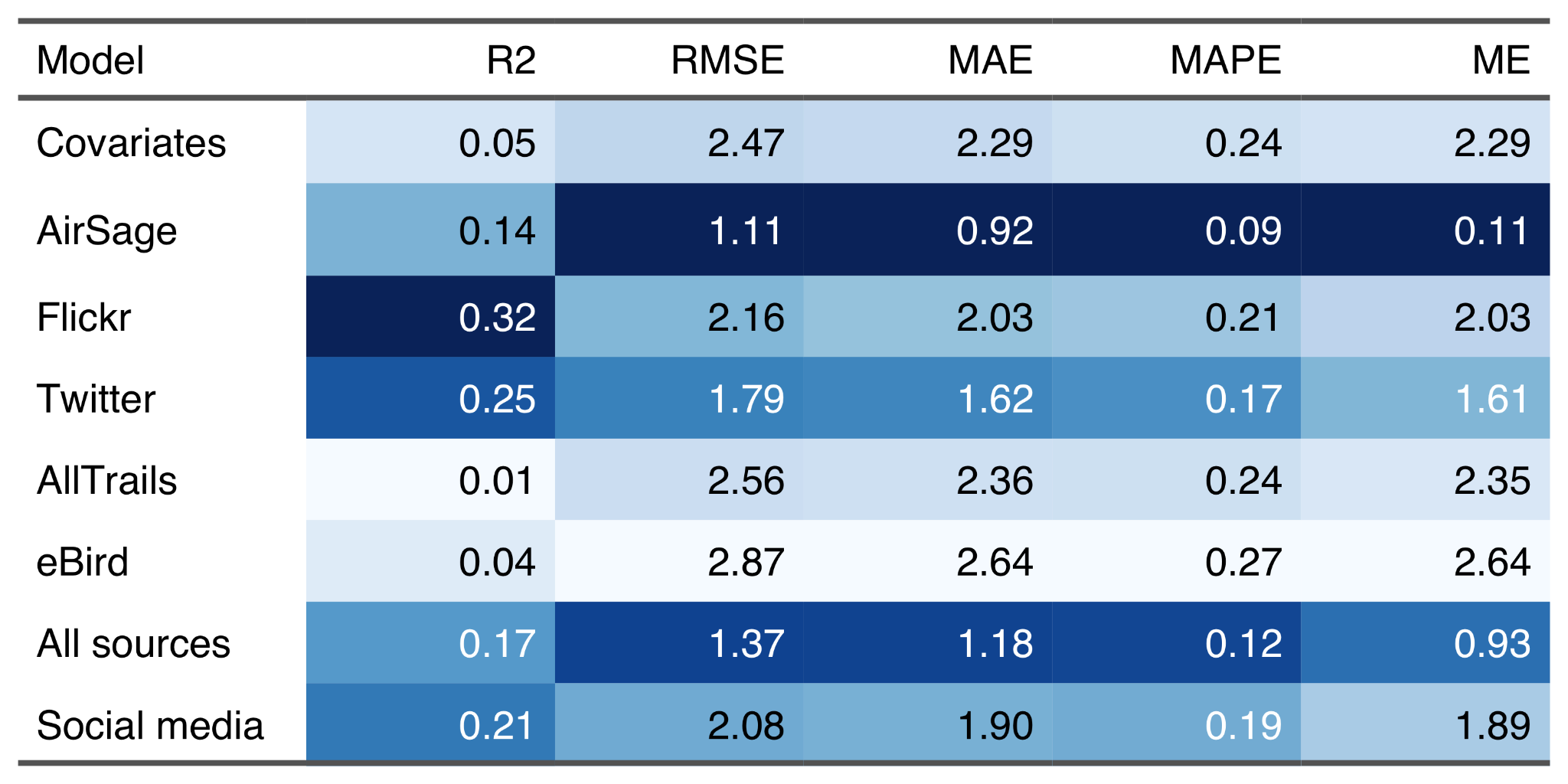


visits


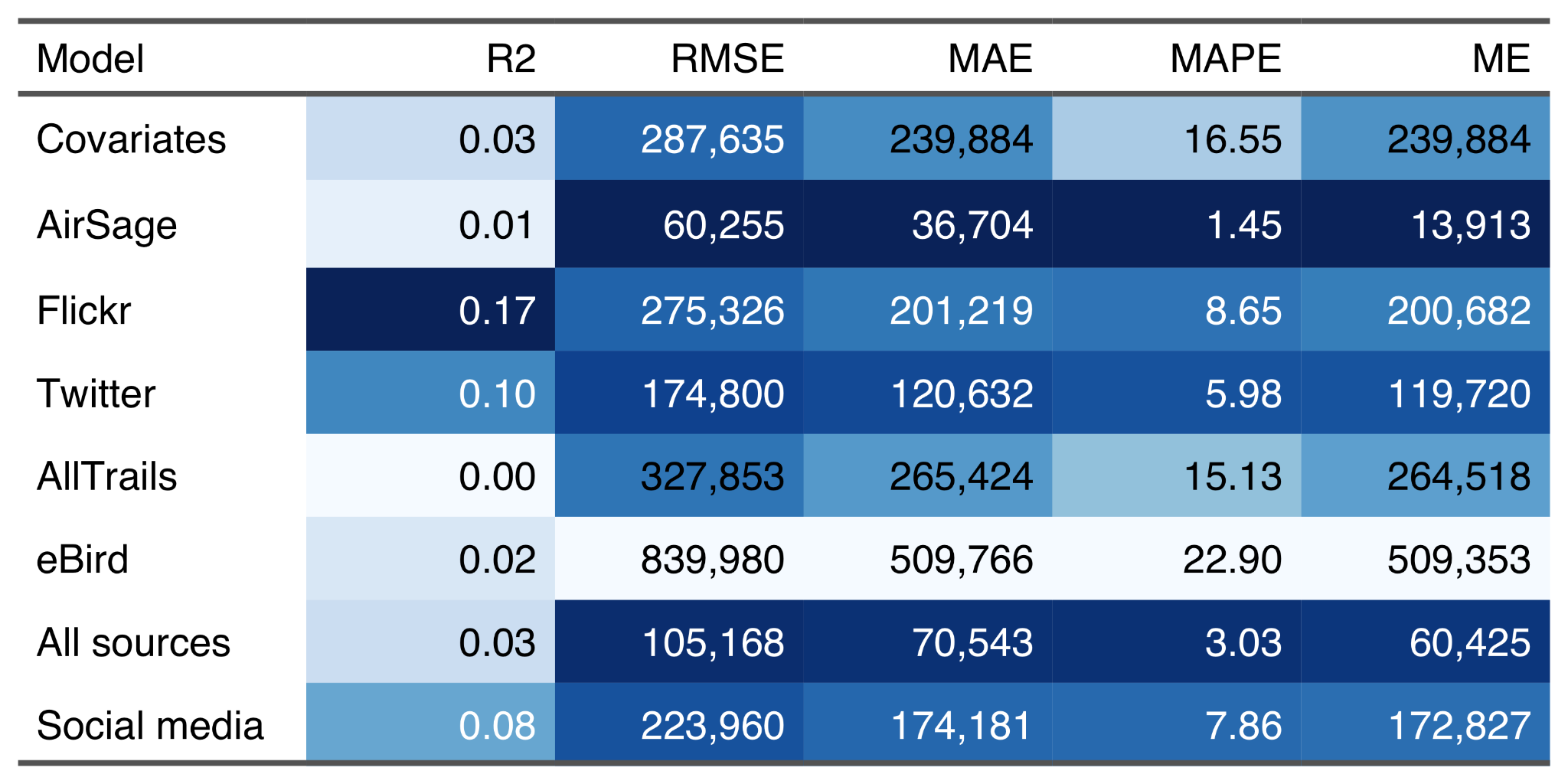


**Table A4**

Full results for CV2

logs


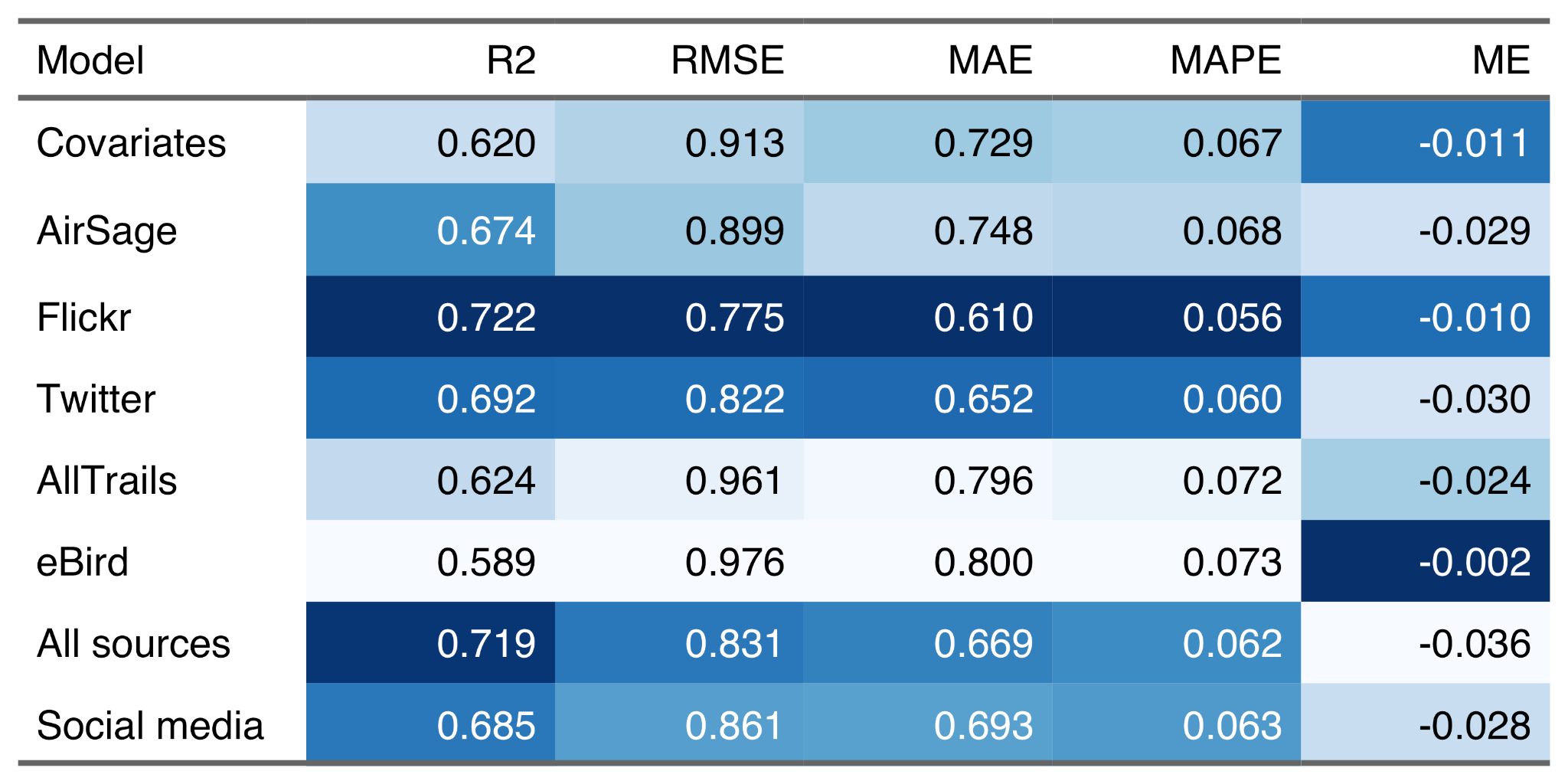


visits


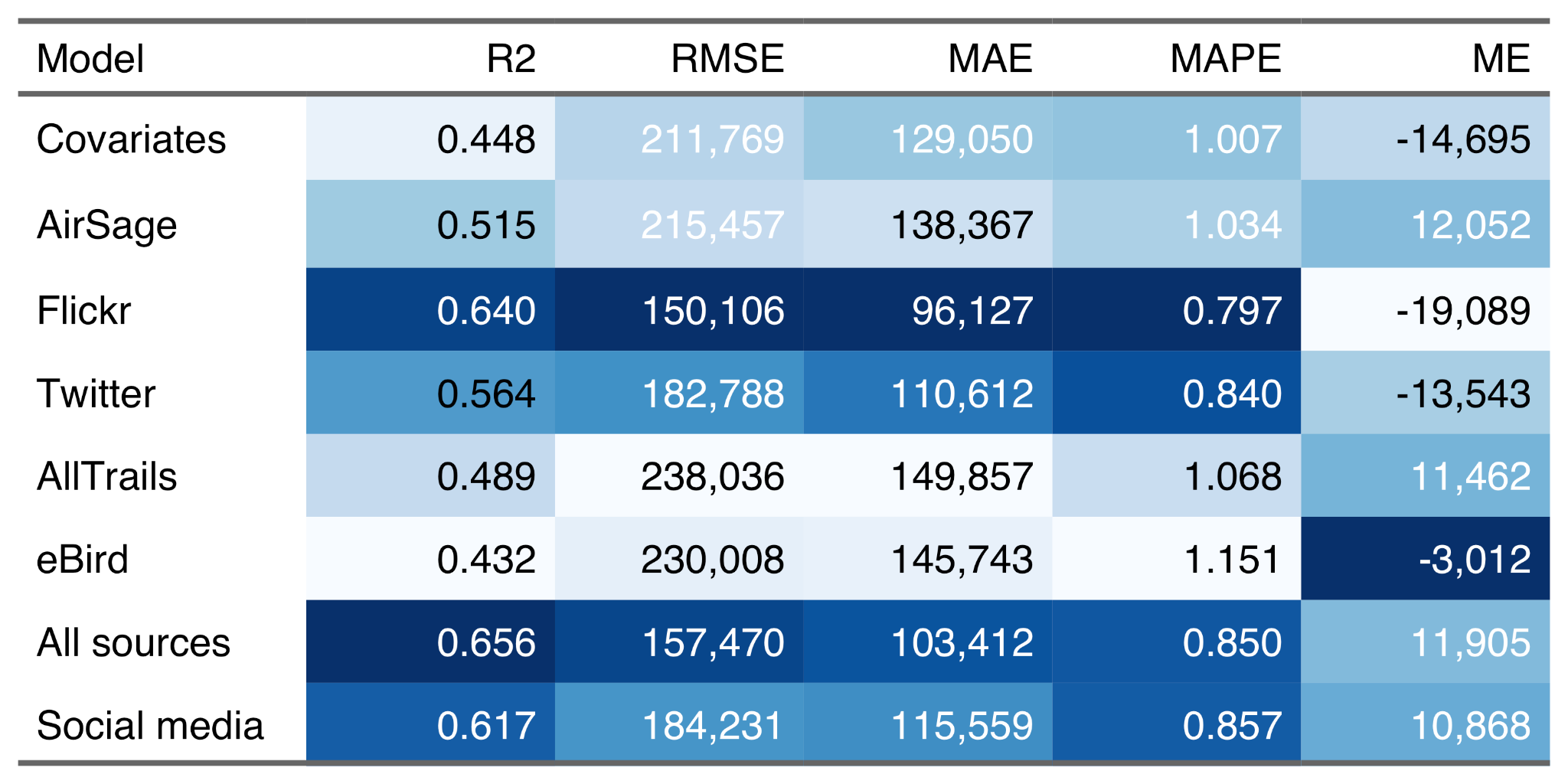


**Table A5**

Full results for CV3

logs


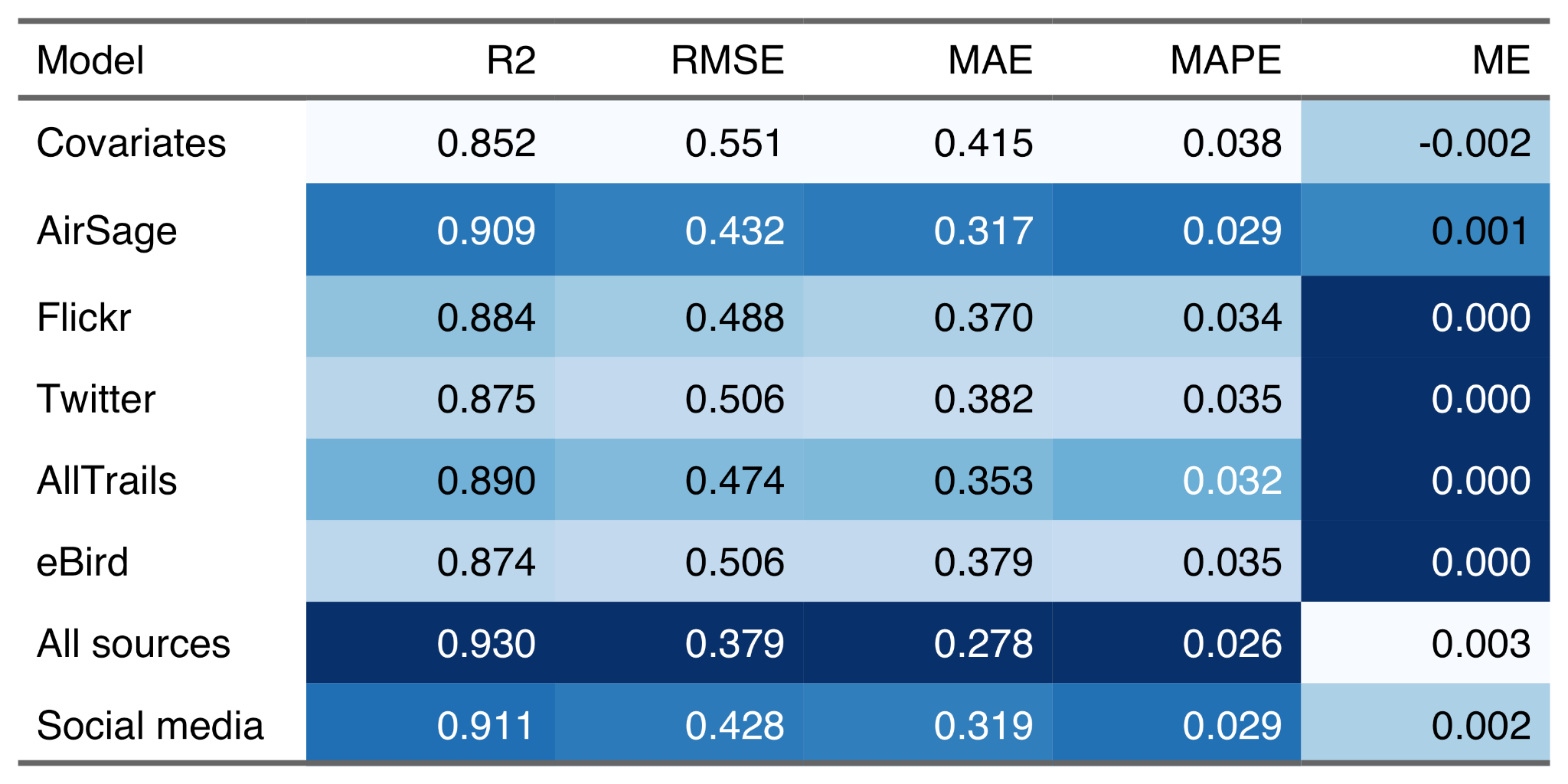


visits


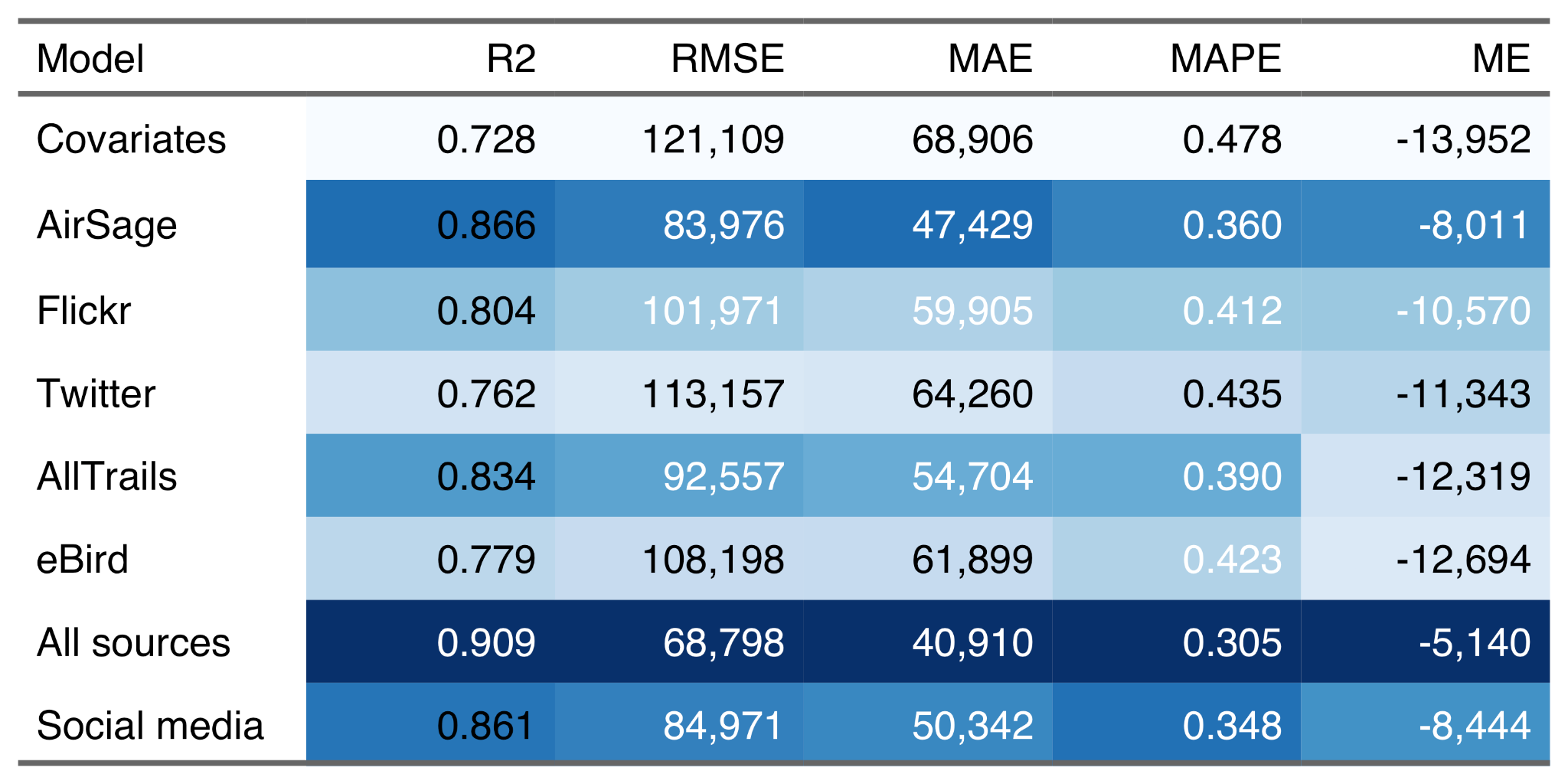


**Table A6:** Pearson’s correlation coefficients between each mobility data source and observed visitation overall and by agency

|  | **AirSage** | **Flickr** | **Twitter** | **AllTrails** | **eBird** |
| --- | --- | --- | --- | --- | --- |
| **Overall** | 0.37 | 0.59 | 0.57 | 0.27 | 0.07 |
| **FWS** | 0.71 | 0.44 | 0.59 | 0.08 | 0.35 |
| **NPS** | 0.35 | 0.43 | 0.30 | 0.15 | 0.03 |
| **USFS** | 0.61 | 0.56 | 0.57 | 0.73 | 0.44 |

**Table A7:** Fixed effects model specification regression results, from fitting the entire dataset. For the factor variables, the references are: Month[1], Year[2018].

|  |  |  |  |
| --- | --- | --- | --- |
| *Predictors* | *Estimates* | *CI* | *p* |
| (Intercept) | 2.82 | 2.12 – 3.52 | **<0.001** |
| AirSage | 0.56 | 0.50 – 0.61 | **<0.001** |
| Flickr | 0.14 | 0.11 – 0.18 | **<0.001** |
| Twitter | 0.17 | 0.13 – 0.20 | **<0.001** |
| AllTrails | 0.18 | 0.14 – 0.22 | **<0.001** |
| eBird | 0.05 | 0.01 – 0.08 | **0.006** |
| temperature °C | 0.01 | 0.00 – 0.02 | **0.003** |
| month [2] | 0.10 | 0.01 – 0.19 | **0.027** |
| month [3] | 0.10 | 0.00 – 0.19 | **0.040** |
| month [4] | 0.17 | 0.06 – 0.28 | **0.003** |
| month [5] | 0.24 | 0.10 – 0.38 | **0.001** |
| month [6] | 0.25 | 0.08 – 0.42 | **0.004** |
| month [7] | 0.30 | 0.11 – 0.49 | **0.002** |
| month [8] | 0.30 | 0.11 – 0.49 | **0.002** |
| month [9] | 0.27 | 0.10 – 0.44 | **0.002** |
| month [10] | 0.24 | 0.11 – 0.37 | **<0.001** |
| month [11] | 0.12 | 0.02 – 0.22 | **0.018** |
| month [12] | 0.07 | -0.02 – 0.17 | 0.120 |
| year [2019] | -0.05 | -0.09 – -0.00 | **0.050** |
| Observations | 1558 | | |
| R^2^ | 0.94 | | |
